# Halogen-containing thiazole orange analogues – new fluorogenic DNA stains

**DOI:** 10.3762/bjoc.13.283

**Published:** 2017-12-28

**Authors:** Aleksey A Vasilev, Meglena I Kandinska, Stanimir S Stoyanov, Stanislava B Yordanova, David Sucunza, Juan J Vaquero, Obis D Castaño, Stanislav Baluschev, Silvia E Angelova

**Affiliations:** 1 Department of Pharmaceutical and Applied Organic Chemistry, Faculty of Chemistry and Pharmacy, Sofia University “St. Kliment Ohridski”, 1 James Bourchier Blvd., 1164 Sofia, Bulgaria; 2 Department of Organic Chemistry and Pharmacognosy, Faculty of Chemistry and Pharmacy, Sofia University “St. Kliment Ohridski”, 1 James Bourchier Blvd., 1164 Sofia, Bulgaria; 3 Departments of Organic and Physical Chemistry, University of Alcala, 28871-Alcala de Henares, Madrid, Spain; 4 Max Planck Institute for Polymer Research, Ackermannweg 10, 55128 Mainz, Germany; 5 Institute of Organic Chemistry with Centre of Phytochemisty, Bulgarian Academy of Sciences, 1113 Sofia, Bulgaria (permanent address)

**Keywords:** cyanine dyes, DFT calculations, green synthesis, nucleic acids, thiazole orange

## Abstract

Novel asymmetric monomeric monomethine cyanine dyes **5a–d**, which are analogues of the commercial dsDNA fluorescence binder thiazole orange (**TO**), have been synthesized. The synthesis was achieved by using a simple, efficient and environmetally benign synthetic procedure to obtain these cationic dyes in good to excellent yields. Interactions of the new derivatives of **TO** with dsDNA have been investigated by absorption and fluorescence spectroscopy. The longest wavelength absorption bands in the UV–vis spectra of the target compounds are in the range of 509–519 nm and these are characterized by high molar absorptivities (63000–91480 L·mol^−1^·cm^−1^). All investigated dyes from the series are either not fluorescent or their fluorescence is quite low, but they become strongly fluorescent after binding to dsDNA. The influence of the substituents attached to the chromophores was investigated by combination of spectroscopic (UV–vis and fluorescence spectroscopy) and theoretical (DFT and TDDFT calculations) methods.

## Introduction

**Scheme 1:**
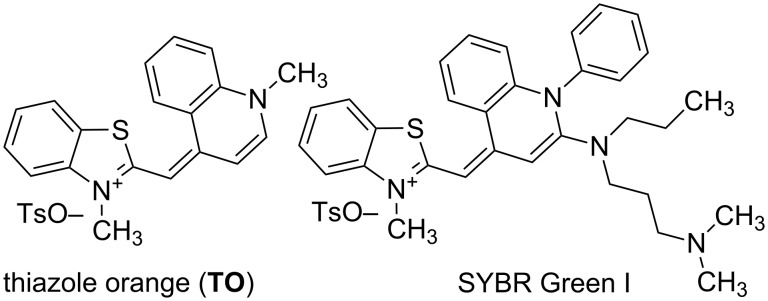
Chemical structures of **TO** and SYBR Green I – commercial monomethine fluorescent dsDNA binders.

Since the discovery by Lee and co-workers [1–2] that the old photographic dye thiazole orange, **TO**, (Scheme 1) has excellent properties as a fluorogenic noncovalent DNA or RNA binder, many representatives of this class of dyes have been developed [3–7].

Thiazole orange does not fluoresce in the free state in solution. Fluorescence appears when the rotation about the monomethine bridge between the two heterocyclic moieties is somehow limited [5–7]. Such a restriction occurs when **TO** derivatives bind to nucleic acids by intercalation between the base pairs [6,8] or presumably between individual bases in single-stranded nucleic acids and in both cases a dramatic increase of the fluorescence is observed. The valuable properties as nucleic acid stains have made these dyes an irreplaceable tool in the active and developing area of bioanalytical chemical research [9–10]. Cyanine dyes have found various bioanalytical applications as soluble DNA intercalators (e.g., in living cell imaging [11] and flow cytometry [12–13], DNA fragment sizing [14–15], as reporter groups in hybridization probes [16–23], single DNA molecule fluorescence microscopy [24–25], gel staining [26], in real-time PCR analysis [27–28]) because of their excellent staining properties. Unsymmetrical cyanines have been conjugated to a variety of molecules, including peptides [29], proteins [30], DNA [17], and DNA analogues such as peptide nucleic acid (PNA) [16,31]. **TO**-based chromophores assembled as a structural scaffold inside nucleic acids (**TO**-tethered nucleic acids) have attracted considerable attention [32]. Hybridization-sensitive fluorescent probes in which **TO** is tethered to a nucleic acid: DNA [22–23,33–36], RNA [20,36] or PNA [18–19,21,31]) strands have been constructed by several research groups (the Krull, Kubista, Seitz and Wagenknecht groups). The continued scientific and commercial interest in the preparation and application of cyanine dyes as bio-probes [37–38] and our research in this area [39–41] has led us to search for novel representatives of this interesting family of compounds. The main goal of the study described here was to investigate the influence of different halogen atoms (connected directly to the chromophore or as side groups) on the molar absorptivity and the fluorescence intensity of a series of new **TO** analogues. Photophysical properties of a series of **TO** analogues that have fluoro- or trifluoromethyl groups connected to the heterocyclic end groups of the chromophores have been studied by Armitage and co-workers [42–43]. According to these studies the fluorination of the symmetrical [42] and unsymmetrical [43] cyanine dyes results in reduced aggregation and significantly improved the photostability and photophysical properties of the dyes.

**Scheme 2:**
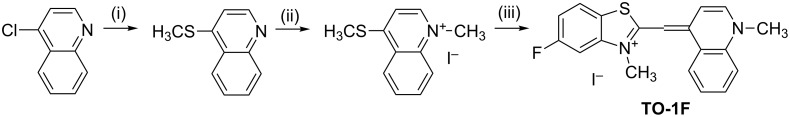
Synthesis of the monofluoro-substituted dye **TO-1F**. Reagents, conditions and yields: (i) sodium methanethiolate (NaSCH_3_), 7 h, reflux; (ii) methyl iodide, overnight, overall yield for (i) and (ii) 9%; (iii) 5-fluoro-2,3-dimethylbenzothiazolium iodide and triethylamine, rt, overnight, yield 24%.

The authors [43] used an interesting modification of Brooker’s method (Scheme 2) for the synthesis of the fluorinated **TO** analogues but they obtained the target compound in very low overall yield. Generally, symmetric or unsymmetric monomethine cyanine dyes can be synthesized by the method of Brooker et al. [44–45] using a condensation reaction between 2-methylthiobenzothiazolium salts with 1-alkyl-4-methylquinolinium salts.

Although Brooker’s synthetic approach usually provides high reaction yields and has very low costs, the method suffers from serious disadvantages. One of these is the possibility of interchange of the alkyl groups at the thioalkyl unit and nitrogen atom in the quaternized 2-alkylthio starting materials, which leads to unexpected reaction products [45–46]. The other major disadvantage is the evolution of methylmercaptan – a toxic pollutant with a very unpleasant odor.

## Results and Discussion

### Synthesis of intermediates and dyes

**Scheme 3:**
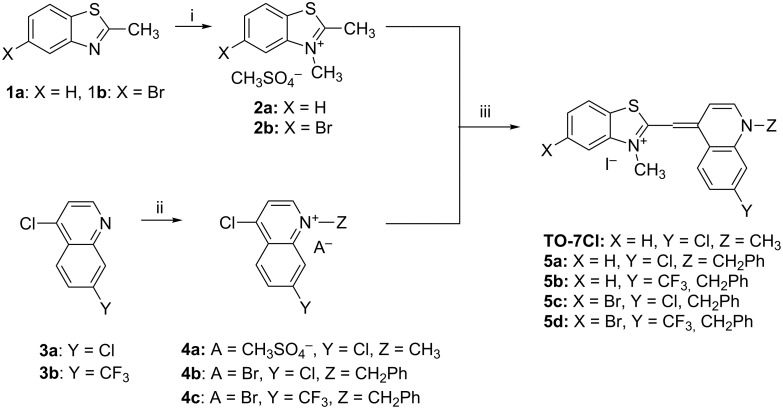
Synthesis of new halogen-containing analogues of **TO**. Reagents, conditions and yields: (i) DMS, 100 °C, 2 h, **2a**: 81%, **2b**: 74%; (ii) DMS or benzyl bromide, 100 °C, 1 h, **4b**: 97%, **4c**: 93%; (iii) EtOH, DIPEA, 2.5 h, rt, **TO-7Cl**: 67%, **5a**: 71%, **5b**: 68%, **5c**: 72%, **5d**: 77%.

On the basis of Green Chemistry concepts [47–49] and with the aim of avoiding the disadvantages of Brooker’s method and reducing the adverse effects of the synthesis, we report here a simple, environmentally more benign (in comparison the Brooker’s method), and efficient synthetic procedure. The method started with the quaternization of all starting materials (Scheme 3), which was performed in a sealed tube under an argon atmosphere to prevent possible side reactions like oxidation of the corresponding *N*-containing heterocylic intermediates or self-condensation of the resulting quaternary products. Carrying out the reaction in bulk without solvents allows scale-up of the quaternary salt synthesis and prevents possible environmental pollution. The known synthetic procedure [50–51] for the target dyes (Scheme 3) was modified to obtain the model compound **TO-7Cl**. The condensation of 2-methylbenzothiazolium salt **2a** and 4,7-dichloroquinolinium methyl sulfate **4a** was conducted in water, methanol or ethanol by stirring the reaction mixture at room temperature or by ultrasound irradiation for not more than 40 minutes.

Prolonged sonication causes heating of the samples in the ultrasonic bath and this makes external cooling difficult. In addition, the ultrasound irradiation promotes side reactions and thus leads to slightly lower reaction yields when compared to the room temperature conditions. It is well known that water is one of the most useful green solvents in organic synthesis. The reaction in water as a solvent provided moderate yields (procedures B1 and B2), but the subsequent scale-up of these reactions could be favorable on using intermediates with better water solubility. The best reaction yields for **TO-7Cl** were obtained on using procedure C1 (Experimental part) with ethanol as the reaction medium. It is worth noting that replacement of the methanol with the environmentally more benign and less harmful ethanol significantly decreases the formation of blue-colored self-condensation byproducts (probably a chromophore constructed from quinolone and quinolinium end groups) and subsequently increases the yield and the purity of the crude products of interest. As mentioned previously, prolonged sonication does not increase the reaction yields, probably due to the promotion of side reactions.

Therefore, the synthesis of the dyes **5a**–**d** was achieved according to procedure C1. In addition, our observation that lower amounts of the blue-colored byproduct were obtained in ethanol solution is probably related to the polarity and the basicity of the medium. We observed that filtration of the final dyes followed by washing the precipitate with ethanol dissolves significant amounts of the byproduct, which was not observed in water or methanol. The subsequent recrystallization of the product from ethanol leads to the isolation of pure (analytical grade) final dyes (**TO-7Cl** and **5a**–**d**). Furthermore, the use of Hünig’s base (*N*,*N*-diisopropylethylamine, DIPEA) is essential for the success of the reaction because of the CH-acidity of the benzyl fragment, which otherwise leads to the possibility of side reactions if trimethylamine or other less hindered basic reagents are employed.

### Photophysical properties

#### Absorption

**Figure 1:**
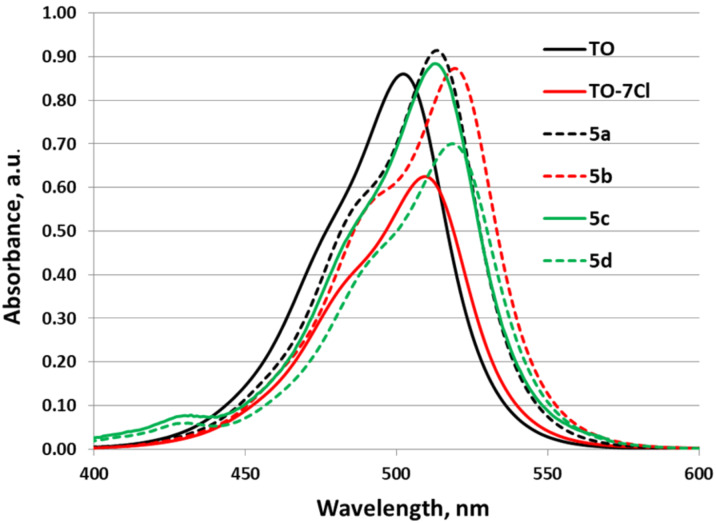
Absorption spectra of the studied compounds in MeOH (*c* = 1 × 10^−5^ M).

The longest wavelength absorption maxima of the studied dyes are in the region 502–519 nm. The corresponding molar absorptivities are in the range of 70000–92000 L·mol^–1^·cm^–1^ (Figure 1 and Table 1). The shape of the absorption bands is typical of **TO**-based chromophores [47–51], with a well pronounced shoulder on the shorter wavelength slope, and it is not sensitive to the type of substituent in the series, thus suggesting similar transition type and geometry of the Franck–Condon excited state.

**Table 1:** Absorption and emission properties of studied dyes in the absence and presence of dsDNA, calculated fluorescence enhancement factor (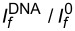) and relative fluorescence quantum yield in presence of dsDNA with respect to **TO**.

Dye	Chemical structure	λ_max_ (ε_max_)^a^	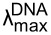 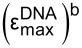	λ_em_^c^	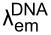 ^c^	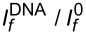	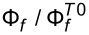 ^d^

**TO**	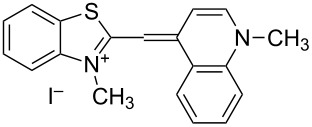	502 (86100)	510 (61100)	549	529	458	1
**TO-7Cl**	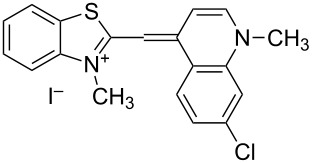	509 (63000)	520 (50500)	550	535	325	0.62
**5a**	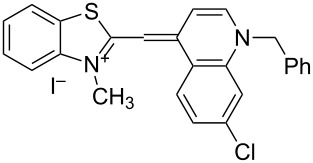	513 (91480)	522 (89600)	620	540	454	1.28
**5b**	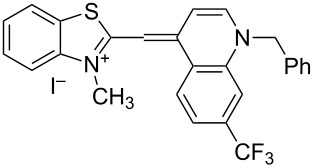	519 (87520)	535 (83200)	627	550	38	0.29
**5c**	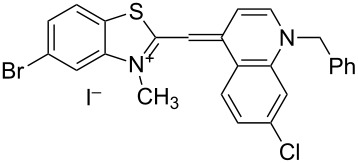	513 (88400)	520 (84900)	600	538	367	0.82
**5d**	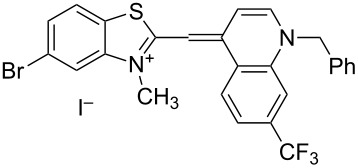	519 (70120)	528 (62100)	602	550	41	0.16

^a^Wavelength of maximum absorbance (nm) and molar absorptivity (L·mol^–1^·cm^–1^) in methanol. ^b^Wavelength of maximum absorbance (nm) and molar absorptivity (L·mol^−1^·cm^−1^) in TE buffer in presence of dsDNA. ^c^Wavelength of maximum fluorescence emission (nm) in absence (λ_em_) and presence (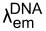) of dsDNA. Excitation wavelength in all cases corresponds to the absorption maximum of the dye–dsDNA complex; ^d^The fluorescence quantum yields in presence of DNA were measured by a gradient method (integrated fluorescence intensity against absorbance) using **TO** as a reference. All measurements were performed under the same experimental conditions (dsDNA concentration, optical setup, etc.). These are relative values, and should be discussed only in terms of comparison with **TO**.

It can be seen from the results in Table 1 that the replacement of a hydrogen atom with an electron-withdrawing group at the C-7 position in the quinoline moiety of the chromophore leads to a bathochromic shift of the longest wavelength absorption maximum in all new compounds when compared to **TO**. The presence of a benzyl substituent in compounds **5a**–**d** doesn’t have significant effect on the maxima position, slightly shifting the transition further towards lower energies when compared to **TO-7Cl** (in the case of **5a** the red shift is 4 nm and can be attributed solely to this effect). The bathochromic shift is more pronounced in **5b** and **5d**, which bear the stronger electron-withdrawing CF_3_ group, when compared to the chloro-substituted analogues. It is also noteworthy that the presence of the Br substituent in the C-5 position of the benzothiazole system does not influence the positions of the absorption maxima (cf. **5a** vs **5c** and **5b** vs **5d**) but does lead to a decrease in the molar absorptivity (up to 20% from **5b** to **5d**).

**Figure 2:**
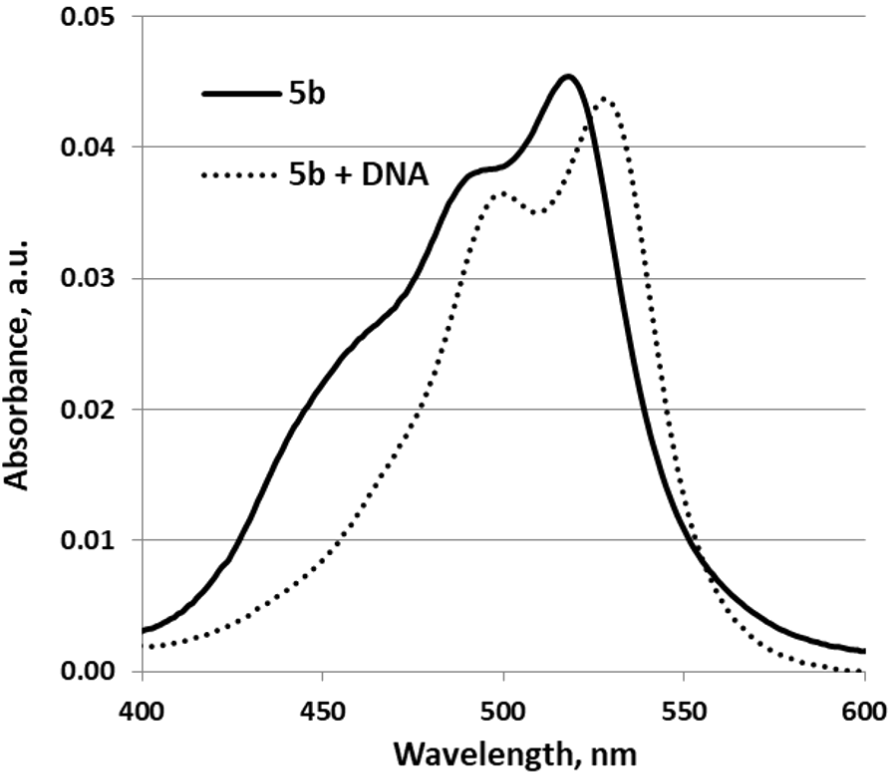
Absorption spectra of dye **5b** (*c* = 5 × 10^−7^ M) in TE buffer, in the absence and presence of dsDNA (*c*_DNA_ = 1.4 × 10^−4^ M).

The addition of dsDNA to the dye solutions in TE buffer led to clear bathochromic (in the range 6 to 10 nm) and hypohromic shifts and this is illustrated as an example for **5b** in Figure 2. Such a spectral change is generally accepted as evidence for the intercalation of dye molecules into DNA [39–41].

#### Emission

**Figure 3:**
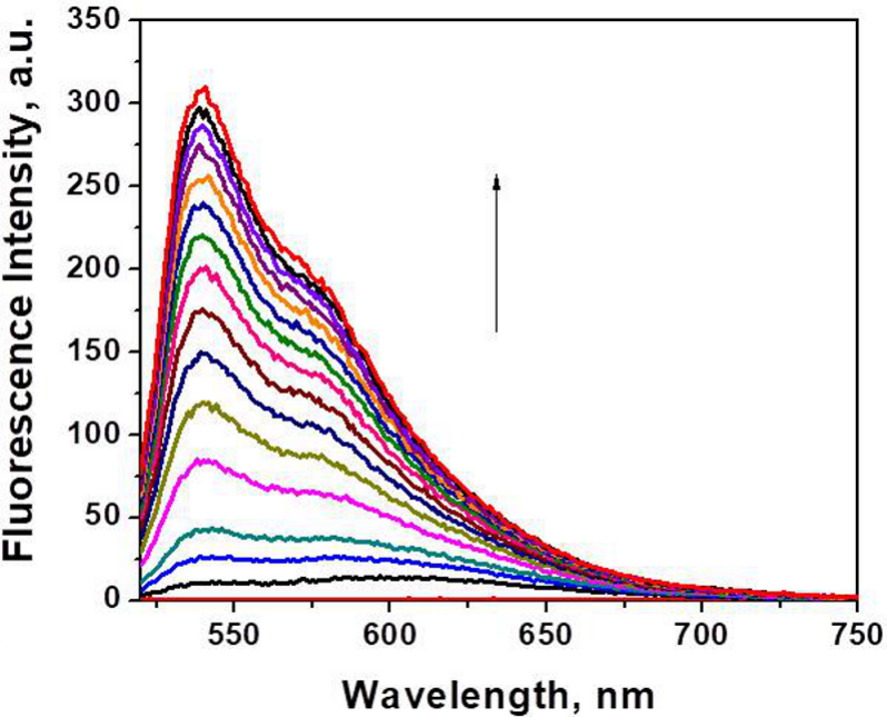
Fluorescence spectra of dye **5a** (*c* = 5 × 10^–7^ M) in TE buffer and in the presence of increasing concentrations of dsDNA (*c* = 0–1.4 × 10^−4^ M).

All dyes have a negligible intrinsic ﬂuorescence in TE buffer, but upon addition of dsDNA a considerable enhancement in ﬂuorescence intensity is observed, even in equimolar ratios of dye/DNA base pairs (Table 1, Figure 3). A strong hypsochromic shift accompanied by a dramatic change in the band shape is observed in the fluorescence maxima of the free dyes in TE buffer upon addition of dsDNA. The fluorescence maxima of dye–dsDNA complexes suggest an increased relative intensity of the 0–0 transition, which further confirms the intercalation of the ligands in all cases studied.

The measured enhancement in the ﬂuorescence intensity upon binding to dsDNA at constant dye concentration (5 × 10^−7^ M) and increasing dsDNA concentration (from 0 to 1.4 × 10^−4^ M) is shown in Figure 3. Comparison of the spectroscopic titration data for the series of dyes revealed that **5a** has the lowest initial intrinsic fluorescence on the one hand and the highest ﬂuorescence intensity in presence of dsDNA on the other, which determines the highest fluorescence enhancement factor of 454 for this compound. The latter value (454) is practically identical to that of the reference **TO** (458), but the fluorescence quantum yield 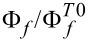 measured for **5a** is twice as high as that of **TO-7Cl**, which can be ascribed to the effect of the benzyl substituent. While the chloro substituent itself leads to a decrease of the relative fluorescent quantum yield (62% for **TO-7Cl** compared to **TO**), the benzyl group in **5a** increases it two fold, making **5a** almost 30% brighter than **TO** under the same conditions (Table 1). In contrast, the dyes bearing trifluoromethyl groups in the 7 position of the quinoline moiety show weak emission even in presence of host molecules. This can be attributed to the strong electron-withdrawing effect of the CF_3_ group, which change the pathways for deactivation of the excited state [43]. The replacement of the hydrogen atom at the C-5 position in the benzothiazole side of the molecules with a bromo substituent does not have any significant influence on the fluorescence of dyes **5c** and **5d**, which is similar to its effect on the absorption spectra and is in contrast to the by Armitage et al. observed influence of the replacement of hydrogen with fluorine atom in the benzothiazole ring [43].

#### Photostability

**Figure 4:**
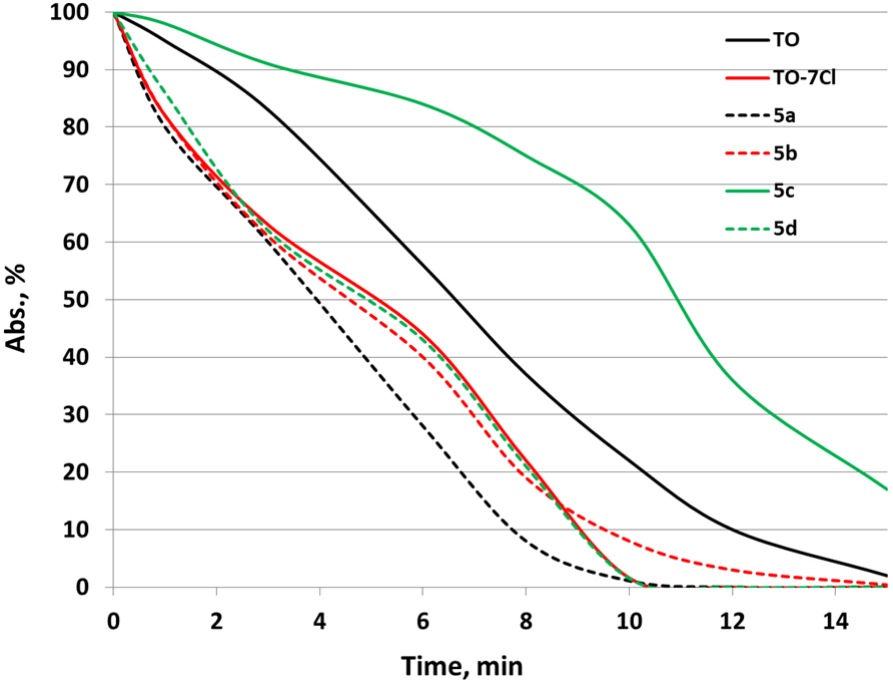
Photostability of dyes **TO-7Cl** and **5a**–**d** in acetonitrile in the concentration range of 1 × 10^−5^ M.

The photostability of all dyes in the series was evaluated in acetonitrile with irradiation at 254 nm with a mercury lamp in equal intervals of two minutes and the results were compared with those obtained for the commercial dye **TO**. All dyes show similar photostability to **TO** except for dye **5c**, which has a much higher photostability than the commercial dye (Figure 4).

All dyes from the series demonstrate much higher photostability upon irradiation at 532 nm by 80 mW continuous wave frequency doubled DPSS (diode pumped solid state) laser, (Figures S21–S24, Supporting Information File 1).

### Computational studies

**Figure 5:**
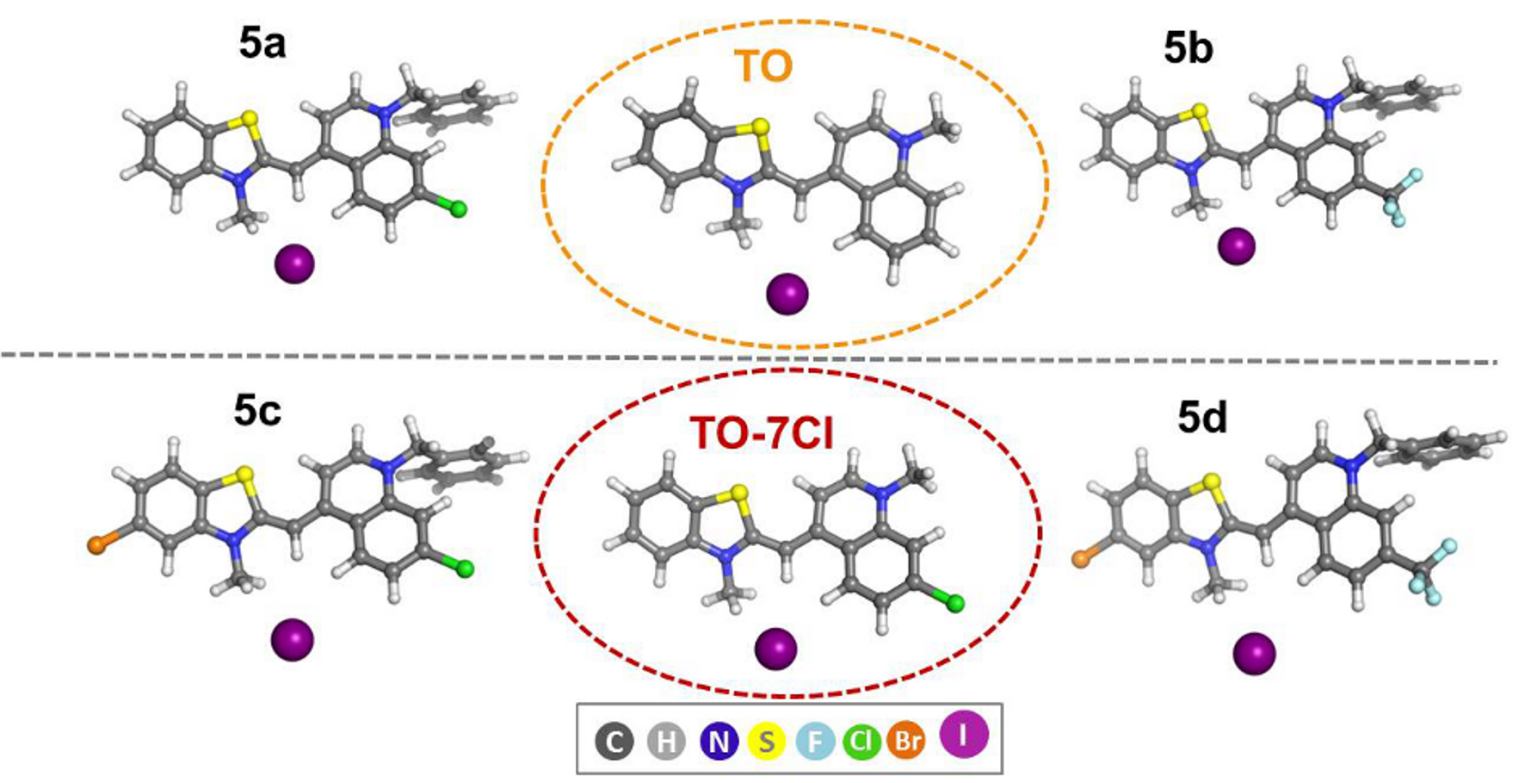
B3LYP optimized structures.

Optimized structures of the preferred conformers of the cationic fragments of all dyes with the counterion I^−^ (in the preffered position) are presented in Figure 5 with the respective atom color scheme.

The electronic reorganizations upon excitation were probed using time-dependent density functional theory (TDDFT) calculations. TDPBE0 (time-dependent density functional theory calculations using Perdew–Burke–Ernzerhof exchange-correlation functional) calculations with the 6–311+G(2d,p) basis set for all atoms except I and with the Stuttgart-Dresden SDD effective core potential (ECP) basis set for I predict two bands for all compounds in the range of 400–500 nm. The calculated optical parameters such as the absorption maximum (λ_max_), oscillator strength (*f*) and frontier orbital energy levels are provided in Table 2. The calculated λ_max_ for the dyes are in the following order: **TO** (452 nm) < **TO-7Cl** (460 nm) = **5a** (460 nm) < **5c** (468 nm) < **5b** (468 nm) < **5d** (481 nm). The bathochromic shift of the longest wavelength maximum upon substitution in the quinolone moiety is consistent with the experimentally observed behavior (**TO-7Cl** compared to **TO**). There is no difference in the predicted maxima for **TO-7Cl** and **5a**, so the experimentally observed 4 nm red shift upon replacement of the methyl by a benzyl group in the same moiety is not manifested in the theoretical calculations. Theoretically calculated absorption maximа for the couples **5a/5c** and **5b/5d** differ by 8 nm and 11 nm, respectively.

**Table 2:** TDDFT/PBE0 excitation energies (eV), wavelengths (nm) (in parentheses), oscillator strength *f*, HOMO and LUMO energies and energy differences (HOMO–LUMO gap) (eV) in methanol for compounds **5a**–**d**, **TO** and **TO-7Cl** in methanol.

Compound	HOMO→LUMO		HOMO-3→LUMO				
	Excitation energy (wavelength)	*f*	Excitation energy (wavelength)	*f*	HOMO	LUMO	HLG

**TO**	2.74 (452)	0.777	2.90 (428)	0.137	−5.99	−2.72	3.27
**TO-7Cl**	2.70 (460)	0.647	2.82 (439)	0.310	−6.03	−2.80	3.23
**5a**	2.70 (460)	0.595	2.83 (437)	0.471	−6.04	−2.81	3.24
**5b**	2.64 (470)	0.327	2.77 (447)	0.703	−6.08	−2.90	3.18
**5c**	2.65 (468)	0.403	2.78 (446)	0.714	−6.10	−2.89	3.21
**5d**	2.58 (481)	0.218	2.73 (454)	0.862	−6.11	−2.98	3.14

**Figure 6:**
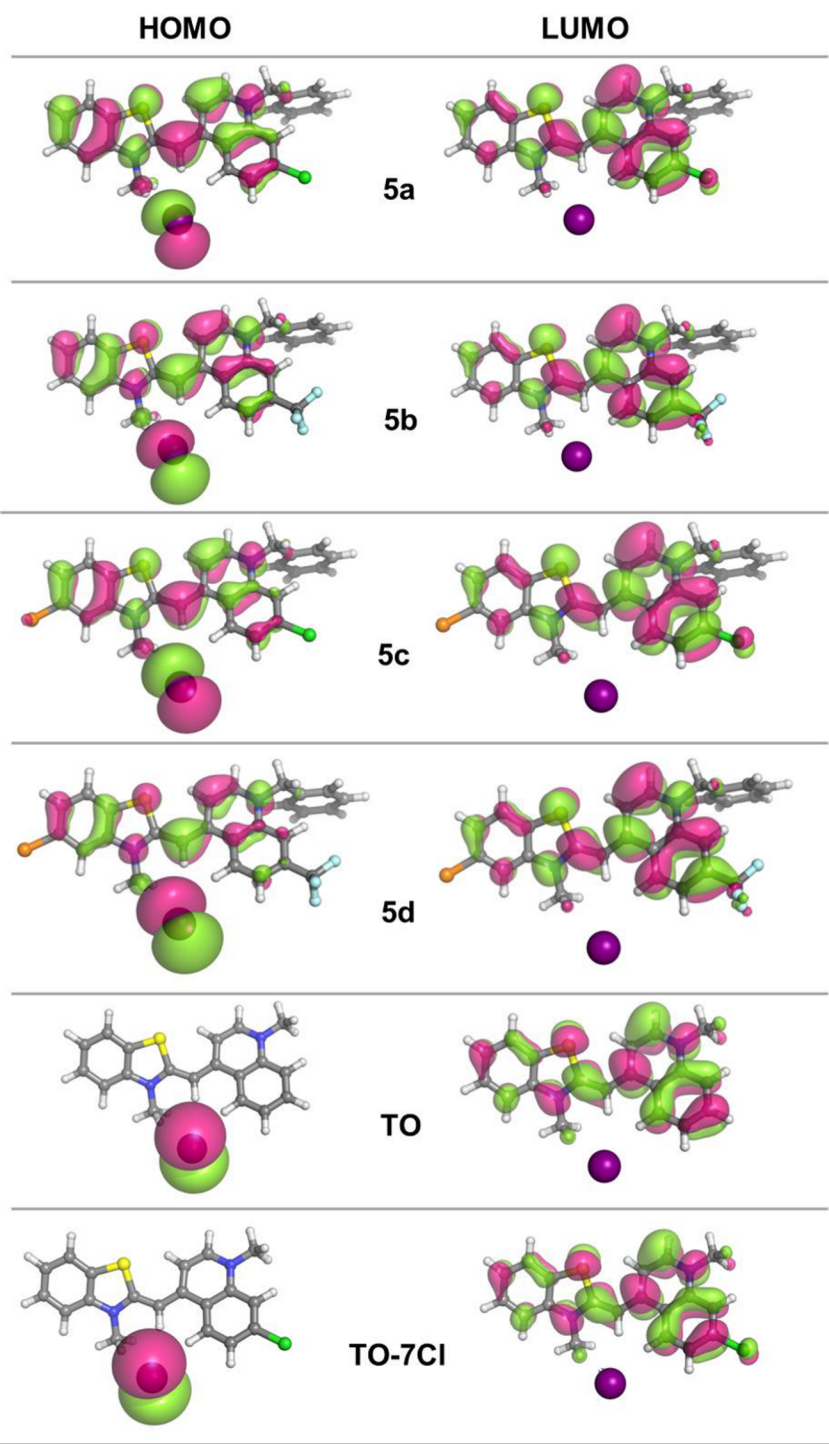
Graphical representation of the frontier orbitals (isodensity plot, isovalue = 0.02 a.u.).

The first excited states are determined by HOMO (highest occupied molecular orbital)→LUMO (lowest unoccupied molecular orbital) transitions with oscillator strengths in the range of 0.218–0.777. The oscillator strengths of the HOMO-1 and HOMO-2 to LUMO transitions are very small (negligible), so the transitions seen in the absorption spectra in the range of 437–454 nm are actually HOMO-3→LUMO transitions. The oscillator strengths of HOMO-3→LUMO transitions are in the range of 0.137–0.862 and for dyes **5b**–**d** these transitions are predicted to be more intense than the HOMO–LUMO ones. The HOMO–LUMO gaps are similar and range from 3.14 eV to 3.27 eV for the compounds in methanol. The frontier orbital energy difference for **TO** is the highest, thus confirming the lower absorption wavelength of **TO**. In compounds **5a**–**d** the HOMO was found to be populated over the entire molecular system except for the benzyl fragment and the substituents in position 7 in the quinolone moiety. The LUMO was found to be delocalized over the quinolinium and, to a lesser extent, the benzothiazolium fragments (Figure 6).

**Figure 7:**
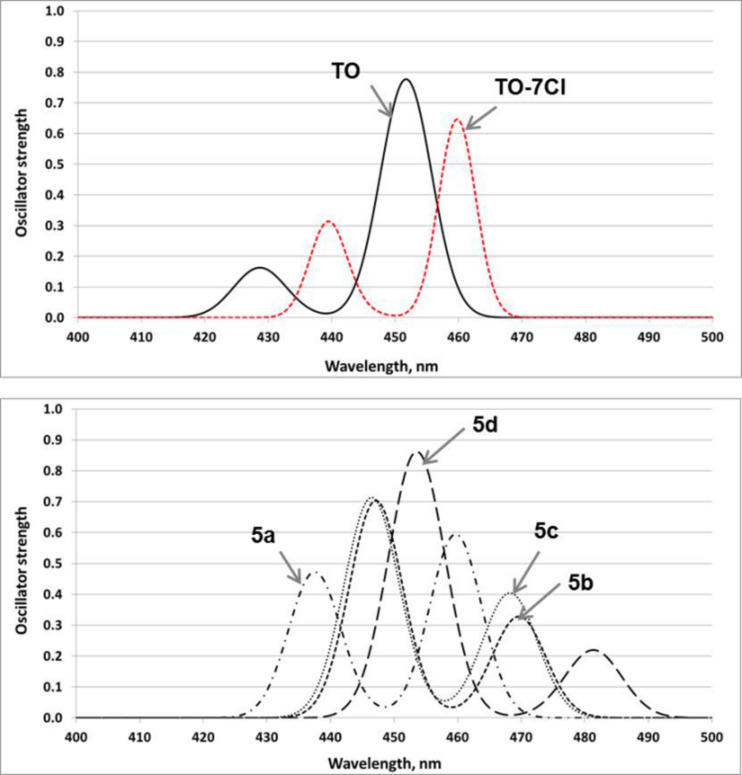
Simulated TDPBE0 spectra in methanol.

The simulated with Doppler broadening with a full width at half-maximum (fwhm) of 0.25 eV and a height proportional to the oscillator strength for each transition spectra (Figure 7) are consistent with those previously reported for cyanine dye monomers [52] and explain the inherent band structure of the monomer’s experimental spectrum with one maximum and a shoulder at shorter wavelength.

**Figure 8:**
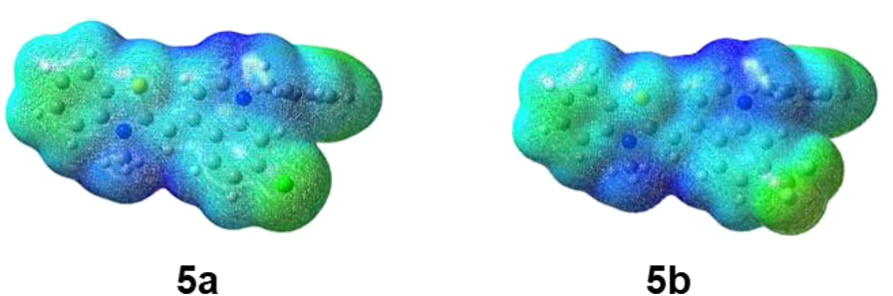
Electron density (isovalue = 0.002) mapped with electrostatic potential (color scheme: green for negative surface map values and blue for the positive ones).

The bathochromic shift observed for compounds **5b** and **5d** (bearing the stronger electron-withdrawing CF_3_ group) is not surprising in view of the higher negative electron density of the cationic species located in this fragment (electron density, mapped with electrostatic potential for **5a** and **5b**, Figure 8).

## Conclusion

Two novel cationic compounds **4b** and **4c** and four asymmetric monomeric monomethine cyanine dyes containing halogen substituents (**5a**–**d**) were synthesized by an environmentally more benign procedure. The properties of the dyes as fluorescence probes for dsDNA detection were investigated and compared with those of the commercial dye **TO**. In TE buffer solution the dyes from the series have negligible fluorescence themselves, but they form strongly fluorescent dye–dsDNA complexes. Apart from dyes **5b** and **5d**, all other dyes demonstrated photophysical properties similar to those of **TO**. One of the new analogues (**5a**) shows higher fluorescence in presence of dsDNA in comparison with **TO** and this property can be used to increase the sensitivity and precision in the appropriate bioanalytical methods. Higher photochemical stability was demonstrated by dye **5c** and this had very low fluorescence in the free state. Computational tools were used in an attempt to explain the optical properties of the studied chromophores. The results are encouraging and warrant further investigations into the newly synthesized dyes as DNA fluorogenic binders. A further study of several aspects (including quantum chemical calculations and experimental studies on the DNA binding mechanism) should be carried out on these dyes in order to exploit in details their promising properties.

## Experimental

### Computational details

The molecular ground state geometries of the cationic fragments of **TO**, **TO**-**7Cl**, **5a–d** with I^−^ counterions were fully optimized using the Becke three-parameter exchange functional with the Lee–Yang–Parr correction (B3LYP) functional [53–54]. We adopted the 6–31+G(d,p) [55–57] basis set for C, H, O, N, F, Cl, and Br and the SDD [58] effective core potential (ECP) basis set basis set for I. *C*_1_ symmetry was assumed for all systems and default convergence criteria were used; local minima were verified by establishing that the Hessians had zero negative eigenvalues. The structures with the counterion were optimized in methanol by means of the integral equation formalism polarizable continuum model (IEFPCM) [59]. TDPBE0 calculations were performed to compute the 20 lowest excited states of each structure [6–311+G(2d,p) basis set for all atoms except I]. The effects of surroundings were included in TDDFT calculations. All calculations were performed using Gaussian 09 [60]. The PyMOL molecular graphics system was used to generate the molecular graphics images [61].

### General

All solvents used in the present work were commercially available (HPLC grade). The starting materials **1a**, **1b**, **3a**, and **3b** were commercially available and were used as supplied. Melting points were determined on a Kofler apparatus and are uncorrected. NMR spectra were obtained on a Bruker Avance III 500 DRX 600 MHz spectrometer in DMSO-*d*_6_. UV–vis spectra were measured on a Unicam 530 UV–vis spectrophotometer and the fluorescence spectra on a Cary Eclipse fluorescence spectrophotometer (Varian, Australia). The GC–MS analysis was performed on a gas chromatograph HP 6890 GC System Plus coupled to a mass selective detector HP 5973 MSD. The mass selective detector operated in electron impact ionization mode at 70 eV electron energy, the ion source temperature was set to 230 °C, and the quadrupole temperature was 15 °C. The mass scan range was 30–750 amu. Compounds **2a** and **4a** were synthesized by methods described in the literature [51,62].

### Synthesis

#### 5-Bromo-2,3-dimethylbenzo[*d*]thiazolium methyl sulfate (**2b**)

5-Bromo-2-methylbenzothiazole (**1b**, 1 mmol) and dimethyl sulfate (DMS, 1.2 mmol) were mixed in a 50 mL sealed tube equipped with a magnetic stirrer. The tube was flushed with argon for 5 min and was sealed. After 2 h of vigorous stirring at 100 °C the reaction mixture was cooled to room temperature and methanol (10 mL) was added. The mixture was heated again until the product had fully dissolved and then cooled down to room temperature. Diethyl ether (30 mL) was added and the resulting precipitate was filtered off and dried in a desiccator. Yield: 74%; mp 80–83 °C; ^1^H NMR (500 MHz, DMSO-*d*_6_) δ 3.16 (s, 3H, CH_3_), 4.18 (s, 3H, NCH_3_), 7.99 (d, ^3^*J*_HH_ = 8.7 Hz, 1H Ar-H), 8.36 (d, ^3^*J*_HH_ = 8.7 Hz, 1H, Ar-H), 8.65 (s, 1H, Ar-H) ppm.

#### 1-Benzyl-4,7-dichloroquinolinium bromide (**4b**)

4,7-Dichloroquinoline (**3a**, 1 mmol) and benzyl bromide (1.2 mmol) were mixed in a 50 mL sealed tube equipped with a magnetic stirrer. The tube was flushed with argon for 5 min and was sealed. After 1 h of vigorous stirring at 100 °C the reaction mixture was cooled to room temperature and acetone (10 mL) was added. The mixture was stirred at room temperature and diethyl ether (30 mL) was added. The resulting precipitate was filtered off and dried in a desiccator. Yield: 97%. The compound is hygroscopic and unstable and its chemical structure was confirmed from the structures of the final dyes.

#### 1-Benzyl-4-chloro-7-(trifluoromethyl)quinolinium bromide (**4c**)

4-Chloro-7-(trifluoromethyl)quinoline (**3b**, 1 mmol) and benzyl bromide (1.2 mmol) were mixed in a 50 mL sealed tube equipped with a magnetic stirrer. The reaction and the work-up were performed in the same way as in the procedure above. Yield: 93%. The compound is hygroscopic and unstable and its chemical structure was confirmed from the structure of the final dye.

#### 7-Chloro-1-methyl-4-((3-methylbenzo[*d*]thiazol-2(3*H*)-ylidene)methyl)quinolin-1-ium iodide (**TO-7Cl**)

**Procedures A1 and A2:** 2,3-Dimethylbenzo[*d*]thiazolium methyl sulfate (**2a**, 1 mmol) and the appropriate 4,7-dichloro-1-methylquinolinium methyl sulfate (**4a**, 1 mmol) were finely ground together in a mortar and the mixture was transferred to a 50 mL Erlenmeyer flask equipped with a magnetic stirrer and a reflux condenser. Methanol (15 mL) and *N*,*N*-diisopropylethylamine (DIPEA, 2.2 mmol) were added. The reaction mixture was vigorously stirred for 2.5 h at room temperature (A1) or sonicated (A2) for 40 min. Diethyl ether (20 mL) was added and the resulting precipitate was filtered off and dissolved in hot methanol (50 mL). To the hot solution was added saturated aqueous potassium iodide (5 mL). The mixture was cooled to room temperature the precipitate was filtered off, washed with methanol (3 × 10 mL) and diethyl ether (10 mL) and dried in a vacuum desiccator. The dye was purified by three recrystallizations from methanol. Yield from reaction at room temperature (Procedure A1): 43% yield from reaction with ultrasound (Procedure A2) 41%; mp 281–283 °C; ^1^H NMR (500 MHz, DMSO-*d*_6_) δ 4.01 (s, 3H, NCH_3_), 4.11 (s, 3H, NCH_3_), 6.87 (s, 1H, CH), 7.27 (d, ^3^*J*_HH_ = 7.2 Hz, 1H, Ar-H), 7.42 (ddd, ^3^*J*_HH_ = 7.9 Hz, ^3^*J*_HH_ = 7.2 Hz, ^4^*J*_HH_ = 0.7 Hz, 1H, Ar-H), 7.61 (ddd, ^3^*J*_HH_ = 8.4 Hz, ^3^*J*_HH_ = 7.3 Hz, ^4^*J*_HH_ = 1.1 Hz, 1H, Ar-H), 7.74–7.78 (m, 2H, Ar-H), 8.04 (d, ^3^*J*_HH_ = 7.2 Hz, 1H, Ar-H); 8.09 (s, 1H, Ar-H), 8.53 (d, ^3^*J*_HH_ = 7.2 Hz, 1H, Ar-H), 8.79 (d, ^3^*J*_HH_ = 9.27 Hz, 1H, Ar-H) ppm; ^13^C NMR DEPT (135 MHz, DMSO-*d*_6_) δ 34.5 (CH_3_) 42.9 (CH_3_), 88.9 (CH), 108.2 (CH), 113.6 (CH), 118.2 (CH), 123.4 (CH), 125.2 (CH), 127.3 (CH), 128.10 (CH), 128.70 (CH), 145.8 (CH) ppm; IR (nujol) ν_max_: 1601, 1490, 1468, 1367, 1321, 1203, 1160, 1115, 915, 880, 765, 725, 625 cm^−1^; GC–MS *m*/*z*: 324 (100%, [M^+^] − CH_3_); elemental analysis (%) for C_19_H_16_ClIN_2_S *M*_w_ = 466.77, calcd for N, 6.00; found: 5.57.

**Procedures B1 and B2: 2a** (1 mmol) and **4a** (1 mmol) were finely ground together and the mixture was transferred to a 50 mL reaction flask. Distilled water (15 mL) and *N*,*N*-diisopropylethylamine (DIPEA, 2.2 mmol) were added. The reaction mixture was vigorously stirred for 2.5 h at room temperature (B1) or sonicated for 40 min (B2). Saturated aqueous potassium iodide (5 mL) was added and the resulting precipitate was filtered off and washed with methanol (30 mL), cold water (30 mL), diethyl ether (10 mL) and dried in a vacuum desiccator. The dye was purified by two recrystallizations from methanol. Yield B1: 40%; yield B2: 31%.

**Procedures C1 and C2: 2a** (1 mmol) and **4a** (1 mmol) were finely ground together in a mortar and the mixture was transferred to a 50 mL Erlenmeyer flask. Ethanol (15 mL) and *N*,*N*-diisopropylethylamine (DIPEA, 2.2 mmol) were added. The reaction mixture was vigorously stirred for 2.5 h at room temperature (C1) or ultra-sonicated for 40 min (C2). Diethyl ether (20 mL) was added and the resulting precipitate was filtered off and dissolved in boiling ethanol (60 mL). To the hot solution was added saturated aqueous potassium iodide (5 mL). The mixture was cooled to room temperature and the resulting precipitate was filtered off and washed with ethanol (20 mL) and diethyl ether (20 mL) and dried in a vacuum desiccator. The dye was purified by two recrystallizations from ethanol. Yield C1: 67%; yield C2: 59%.

#### Synthesis of monocationic monomethine cyanine dyes **5a–d**

2,3-Dimethylbenzo[*d*]thiazolium methyl sulfate (**2a**, 1 mmol) or 5-bromo-2,3-dimethylbenzo[*d*]thiazolium methyl sulfate (**2b**) and the appropriate 4-chloro-substituted quinolinium intermediate **4a** or **4b** (1 mmol) were synthesized and purified according to procedure C1. The dyes were purified by multiple recrystallizations from ethanol.

**2-((1-Benzyl-7-chloroquinolin-4(1*H*)-ylidene)methyl)-3-methylbenzo[*d*]thiazol-3-ium iodide (5a):** Yield 71%; mp 288–290 °C; ^1^H NMR (500 MHz, DMSO-*d*_6_) δ 4.13 (s, 3H, NCH_3_), 5.94 (s, 2H, NCH_2_Ph), 7.05 (s, 1H, CH), 7.33–7.35 (m, 3H, Ar-H), 7.39–7.42 (m, 2H, Ar-H), 7.45 (d, ^3^*J*_HH_ = 6.9 Hz, 1H, Ar-H), 7.52 (dd, ^3^*J*_HH_ = 7.0 Hz, ^4^*J*_HH_ = 0.9 Hz, 1H, Ar-H), 7.69 (dd, 1H, Ar-H), 7.91 (d, ^3^*J*_HH_ = 7.5 Hz, 1H, Ar-H), 7.92 (d, ^3^*J*_HH_ = 8.1 Hz, 1H, Ar-H), 8.15 (d, ^3^*J*_HH_ = 7.8 Hz, 1H, Ar-H), 8.22 (s, 1H, Ar-H), 8.78 (d, ^3^*J*_HH_ = 6.9 Hz, 1H, Ar-H), 9.00 (d, ^3^*J*_HH_ = 8.9 Hz, 1H, Ar-H) ppm; ^13^C NMR DEPT (135 MHz, DMSO-*d*_6_) δ 33.8, 56.0, 89.5, 107.6, 113.2, 122.6, 123.9, 124.7, 126.1, 127.2, 127.7, 127.9, 128.5, 134.7, 136.5, 139.8, 144.9 ppm; IR (nujol) ν_max_: 1602, 1495, 1450, 1370, 1315, 1110, 974, 780, 750, 725 cm^−1^; GC–MS (*m*/*z*): 324 (100%, [M^+^] − CH_2_Ph); elemental analysis (%) for C_25_H_20_ClIN_2_S *M*_w_ = 542.86, calcd for N 5.16; found: 5.53.

**2-((1-Benzyl-7-(trifluoromethyl)quinolin-4(1*H*)-ylidene)methyl)-3-methylbenzo[*d*]thiazol-3-ium iodide (5b):** Yield 68%; mp 284–285 °C; ^1^H NMR (500 MHz, DMSO-*d*_6_) δ 4.13 (s, 3H, NCH_3_), 5.94 (s, 2H, NCH_2_Ph), 7.06 (s, 1H, CH), 7.34–7.36 (m, 3H, Ar-H), 7.39–7.43 (m, 2H, Ar-H), 7.49 (d, 1H, ^3^*J*_HH_ = 6.5 Hz, Ar-H), 7.52 (dd, *J*^3^_HH_ = 6.8 Hz, ^4^*J*_HH_ = 1.0 Hz, 1H, Ar-H), 7.69 (dd, *J*^3^_HH_ = 6.5 Hz, ^4^*J*_HH_ = 0.7 Hz, 1H, Ar-H), 7.88–7.90 (m, 2H, Ar-H), 7.91 (d, ^3^*J*_HH_ = 7.1 Hz, 1H, Ar-H), 8.15 (d, ^3^*J*_HH_ = 7.1 Hz, 1H, Ar-H), 8.22 (s, 1H, Ar-H), 8.77 (d, ^3^*J*_HH_ = 6.4 Hz, 1H, Ar-H), 9.00 (d, ^3^*J*_HH_ = 7.7 Hz, 1H, Ar-H) ppm; ^13^C NMR DEPT (135 MHz, DMSO-*d*_6_) δ 34.9 (CH_3_) 57.1 (CH_2_), 90.6 (CH), 108.7 (CH), 114.3 (CH), 116.4 (CH), 116.5 (CH), 122.4 (CH), 122.3 (CH), 123.6 (CH), 125.8 (CH), 127.3 (CH), 128.3 (CH), 128.8 (CH), 129.0 (CH), 129.6 (CH), 146.0 (CH) ppm; IR (nujol) ν_max_: 1602, 1503, 1450, 1455, 1380, 1310, 1210, 1155, 1120, 1095, 860, 790, 745, 735, 720, 545 cm^−1^; GC–MS (*m*/*z*): 358 (100%, [M^+^] − CH_2_Ph); elemental analysis (%) for C_26_H_20_F_3_IN_2_S *M*_w_ = 576.42, calcd for N, 4.86; found: 5.20.

**2-((1-Benzyl-7-chloroquinolin-4(1*H*)-ylidene)methyl)-5-bromo-3-methylbenzo[*d*]thiazol-3-ium iodide (5c):** Yield 72%; mp = 279–281 °C; ^1^H NMR (500 MHz, DMSO-*d*_6_) δ 4.03 (s, 3H, NCH_3_), 5.89 (s, 2H, NCH_2_), 6.94 (s, 1H, CH), 7.33 (d, ^3^*J*_HH_ = 7.4 Hz, 2H, Ar-H), 7.36 (d, ^3^*J*_HH_ = 7.2 Hz, 1H, Ar-H), 7.41 (dd, *J*^3^_HH_ = 7.5 Hz, ^3^*J*_HH_ = 1.6 Hz, 2H, Ar-H), 7.43 (s, 1H, Ar-H), 7.60 (dd, ^3^*J*_HH_ = 8.4 Hz, ^3^*J*_HH_ = 1.5 Hz, 1H, Ar-H), 7.72 (dd, ^3^*J*_HH_ = 9.1 Hz, ^4^*J*_HH_ = 1.7 Hz, 1H, Ar-H), 8.04 (d, ^3^*J*_HH_ = 8.4 Hz, 1H, Ar-H), 8.09 (d, ^3^*J*_HH_ = 8.0 Hz, 1H, Ar-H, 8.093 (d, ^3^*J*_HH_ = 7.1 Hz, 1H, Ar-H), 8.79 (d, ^3^*J*_HH_ = 7.3 Hz, 1H, Ar-H), 8.83 (d, ^3^*J*_HH_ = 9.2 Hz, 1H, Ar-H) ppm; ^13^C NMR DEPT (135 MHz, DMSO-*d*_6_) δ 34.8 (CH_3_), 57.4 (CH_2_), 89.9 (CH), 108.8 (CH), 116.6 (CH), 118.3 (CH), 125.0 (CH), 125.1 (CH), 127.2 (CH), 127.5 (CH), 127.9 (CH), 128.6 (CH), 128.8 (CH), 128.8 (CH), 129.6 (CH), 145.9 (CH) ppm; IR (nujol) ν_max_: 1600, 1520, 1450, 1455, 1375, 1325, 1280, 1210, 1150, 1120, 885, 865, 790, 725, 610, 550, 401 cm^−1^; GC–MS (*m*/*z*): 480 (100%, [M^+^] – CH_2_Ph); elemental analysis (%) for C_25_H_19_BrClIN_2_S *M*_w_ = 621.76, calcd for N, 4.51; found: 4.95.

**2-((1-Benzyl-7-(trifluoromethyl)quinolin-4(1*H*)-ylidene)methyl)-5-bromo-3-methylbenzo[*d*]thiazol-3-ium iodide (5d):** Yield 77%; mp = 298 °C dec.; ^1^H NMR (500 MHz, DMSO-*d*_6_) δ 4.02 (s, 3H, NCH_3_), 4.09 (s, 2H, NCH_2_), 5. 98 (s, 1H, CH), 6.71 (s, 1H, Ar-H), 7.04 (s, 1H, Ar-H), 7.34–7.36 (m, 3H, Ar-H), 7.39–7.42 (m, 1H, Ar-H), 7.47 (d, ^3^*J*_HH_ = 7.3 Hz, 1H, Ar-H), 7.65–7.69 (m, 1H, Ar-H), 7.95 (d, ^3^*J*_HH_ = 8.5 Hz, 1H, Ar-H), 8.08 (d, ^3^*J*_HH_ = 8.4 Hz, 1H, Ar-H), 8.16–8.19 (m, 2H, CH), 8.27 (s, 1H, Ar-H), 8.86 (d, ^3^*J*_HH_ = 7.4 Hz, 1H, Ar-H), 9.03 (d, ^3^*J*_HH_ = 8.8 Hz, 1H, Ar-H) ppm; ^13^C NMR DEPT (135 MHz, DMSO-*d*_6_) δ 35.00 (CH_3_), 57.4 (CH_2_), 84.1 (CH), 90.7 (CH), 109.3 (CH), 117.0 (CH), 117.3 (CH), 125.2 (CH), 125.6 (CH), 126.1 (CH), 126.4 (CH), 127.3 (CH), 128.1 (CH), 128.9 (CH), 129.6 (CH), 146.4 (CH) ppm; IR (nujol) ν_max_: 1602, 1515, 1460, 1455, 1365, 1325, 1220, 1090, 875, 805, 790, 740, 725, 560, 530, 401 cm^−1^; GC–MS (*m*/*z*): 438 (100%, [M^+^] – CH_2_Ph); elemental analysis (%) for C_26_H_19_BrF_3_IN_2_S *M*_w_ = 655.31, calcd for N, 4.27; found: 4.52.

## Supporting Information

File 1 Characterisation data for the compounds: NMR and GS–MS spectra; validation of the theoretical computations; photostabilities at 532 nm.
