# Supplementary material for: Halogen-containing thiazole orange analogues – new fluorogenic DNA stains
Source: Beilstein J Org Chem. 2017 Dec 28;13:2902–14. doi: 10.3762/bjoc.13.283 (PMC5753173; doi:10.3762/bjoc.13.283)
Supplement: File 1 — Characterisation data for the compounds: NMR and GS–MS spectra; validation of the theoretical computations; photostabilities at 532 nm. [file Beilstein_J_Org_Chem-13-2902-s001.pdf]

Supporting Information

for

Halogen-containing thiazole orange analogues – new fluorogenic DNA stains

Aleksey A. Vasilev*[1], Meglena I. Kandinska[2], Stanimir S. Stoyanov[2], Stanislava B. Yordanova[2], David Sucunza[3], Juan J. Vaquero[3], Obis D. Castaño[3], Stanislav Baluschev[4] and Silvia E. Angelova*[3,5]

Address: [1] Department of Pharmaceutical and Applied Organic Chemistry, Faculty of Chemistry and Pharmacy, Sofia University “St. Kliment Ohridski”, 1 James Bourchier Blvd., 1164 Sofia, Bulgaria, [2]Department of Organic Chemistry and Pharmacognosy, Faculty of Chemistry and Pharmacy, Sofia University “St. Kliment Ohridski”, 1 James Bourchier Blvd., 1164 Sofia, Bulgaria, [3]Departments of Organic and Physical Chemistry, University of Alcala, 28871-Alcala de Henares, Madrid, Spain, [4]Max Planck Institute for Polymer Research, Ackermannweg 10, 55128 Mainz, Germany and [5]Institute of Organic Chemistry with Centre of Phytochemisty, Bulgarian Academy of Sciences, 1113, Sofia, Bulgaria (permanent address)

Email: Aleksey A. Vasilev - ohtavv@chem.uni-sofia.bg; Silvia E. Angelova - sea@orgchm.bas.bg

*Corresponding author

Characterisation data for the compounds: NMR and GS–MS spectra; validation of the theoretical computations; photostabilities at 532 nm

Table of contents:

Figures S1–S13: characterisation data for the new compounds: copies of ^{1}H and ^{13}C DEPT135 NMR spectra;

Figures S14–S18: copies of GC–MS spectra;

Figure S19: superimposed structures of 3-(4-((3-methyl-1,3-benzothiazol-2(3*H*)-ylidene)methyl)-quinolinium-1-yl)propanoate tetrahydrate (CSD entry OVUJUL / CCDC number 739300)[S1] and B3LYP/6-31+G** optimized structure of 3-(4-((3-methyl-1,3-benzothiazol-2(3*H*)-ylidene)methyl)quinolinium-1-yl)propanoate;

Figure S20: labels assigned to selected atoms in 3-(4-((3-methyl-1,3-benzothiazol-2(3*H*)-ylidene)methyl)quinolinium-1-yl)propanoate;

Table S1: Experimental structural data (selected bond lengths and angles) from Ref. [S1] compared to the B3LYP/6-31+G** calculated values for the fully optimized structure of 3-(4-((3-methyl-1,3-benzothiazol-2(3*H*)-ylidene)methyl)quinolinium-1-yl)propanoate without crystallization water molecules.

Figures S21–S24: Photostabilities of **TO**, **TO-7Cl**, **5a**, **5d** measured by a continuous wave frequency doubled DPSS (diode pumped solid state) - laser, operating at λ = 532 nm. The maximal laser power is 80 mW. Via a ND (neutral density) - filter wheel (NDC-100S-4M, Thorlabs Inc.) the laser power can be tuned in broad range. The laser output via couple of cylindrical and spherical lenses is collimated to a nearly rectangular spot with dimensions of 20 mm x 40 mm, thus the whole entrance aperture of the 10 mm square cuvette is covered. The maximal excitation intensity was 10 mWcm^{-2}, with a possibility for controllable decrease.

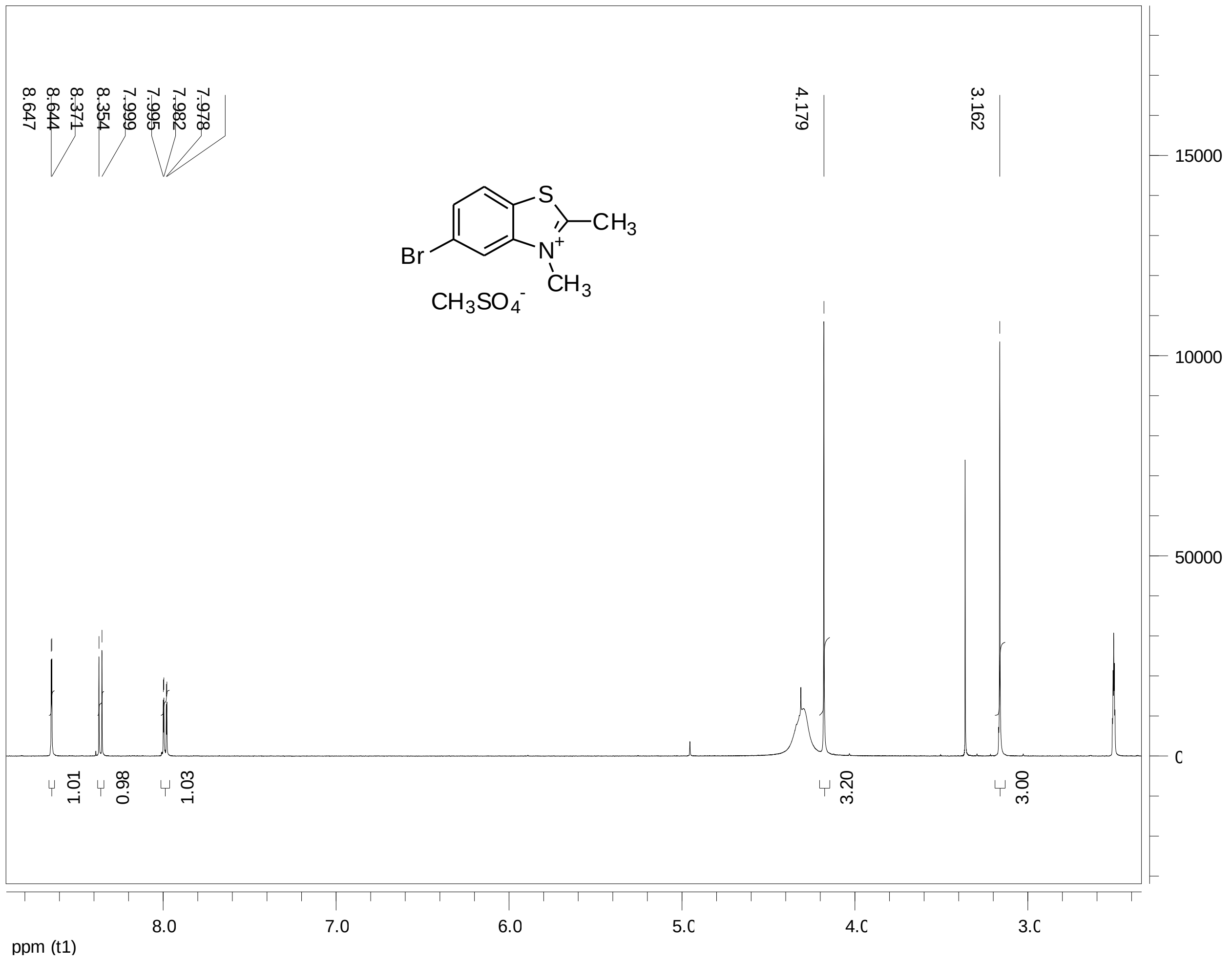

Figure S1: ^{1}H NMR spectrum of **2b**.

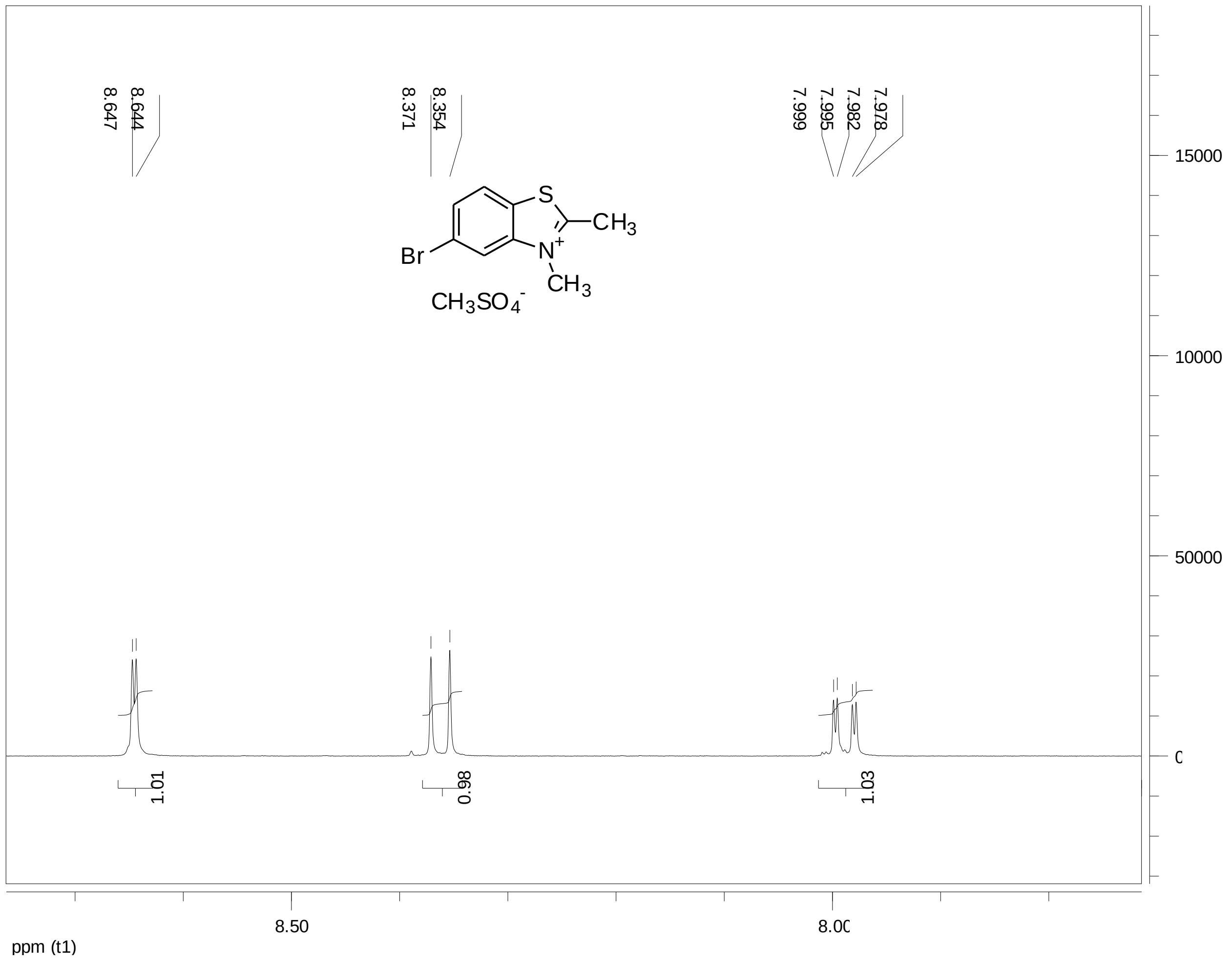

Figure S2: ^{1}H NMR spectrum of **2b** (7.5–9.0 ppm).

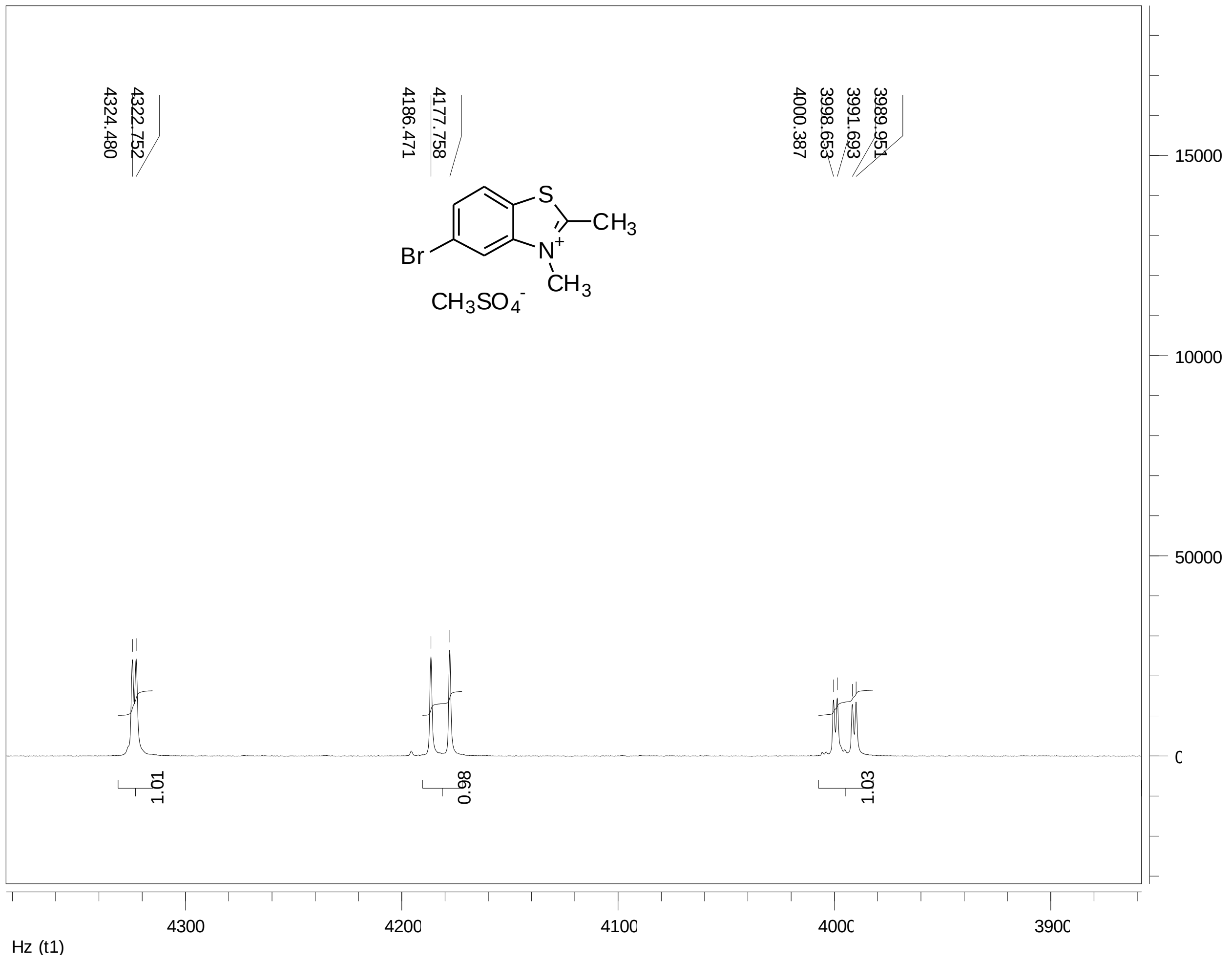

Figure S3: ^{1}H NMR spectrum of **2b** (in Hz) (7.5–9.0 ppm).

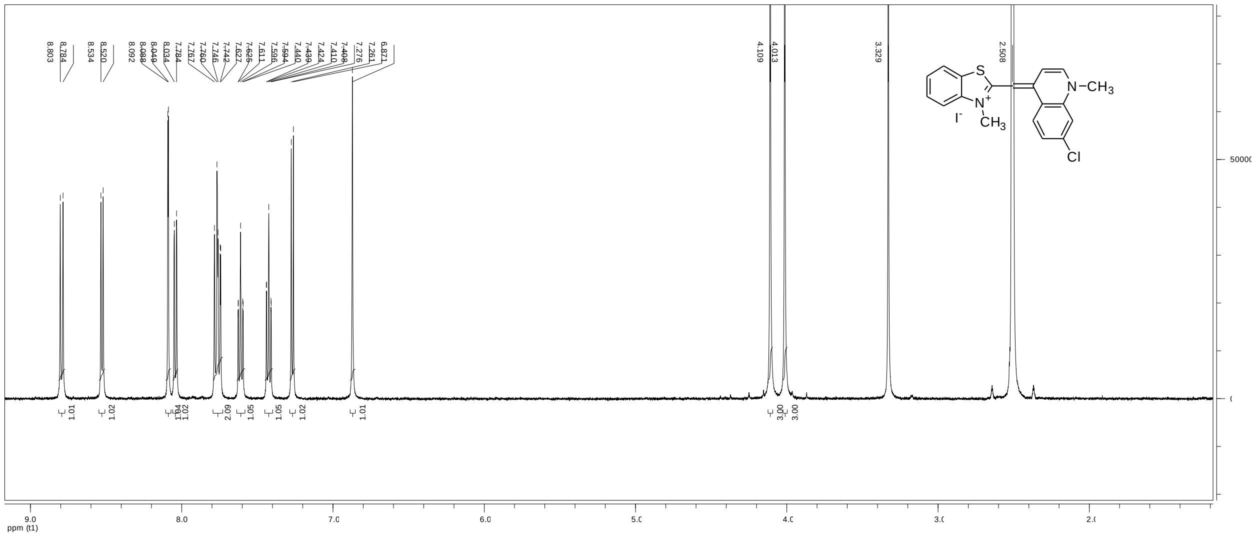

Figure S4: ^{1}H NMR spectrum of **TO-7Cl**.

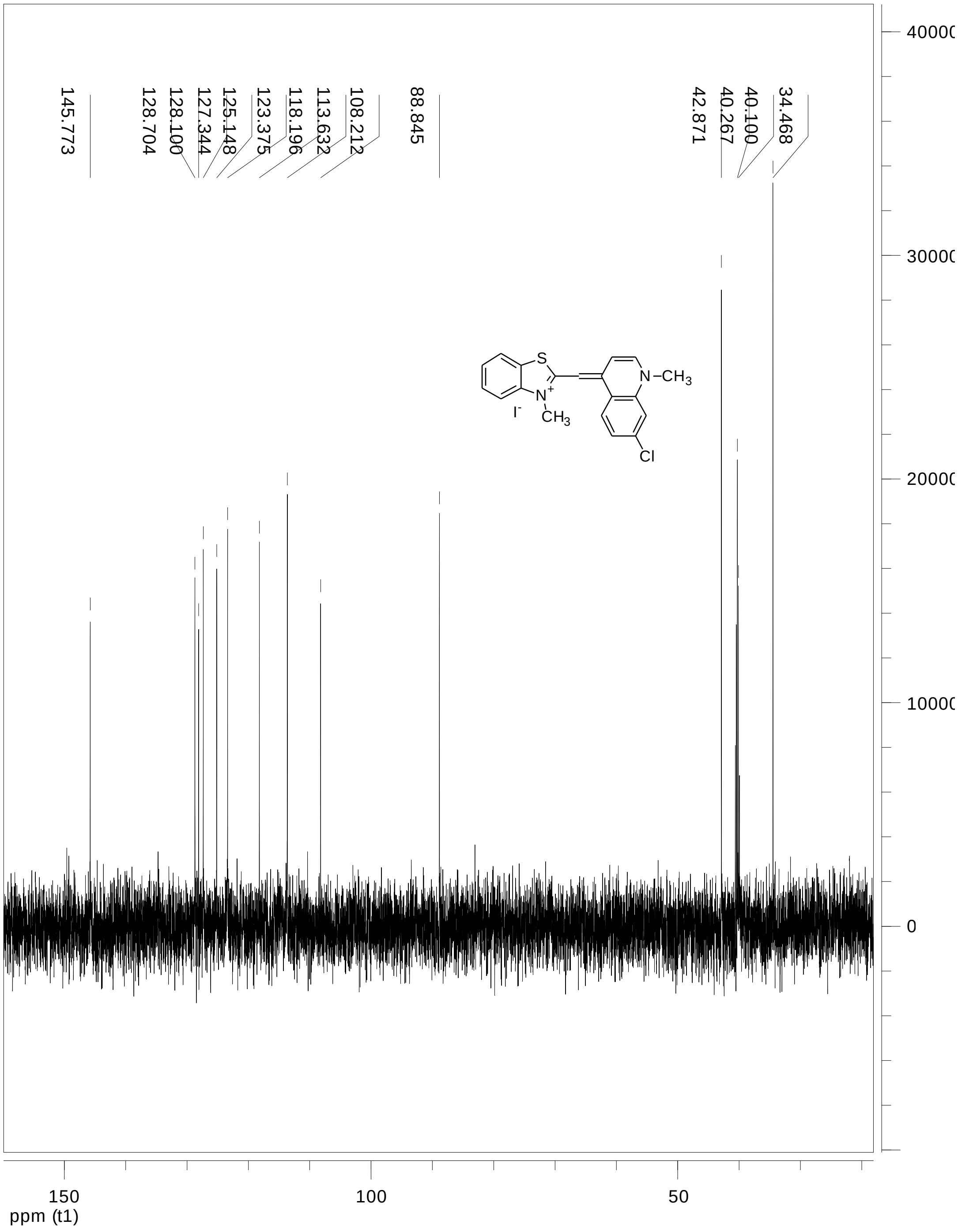

Figure S5: DEPT135 spectrum of **TO-7Cl**.

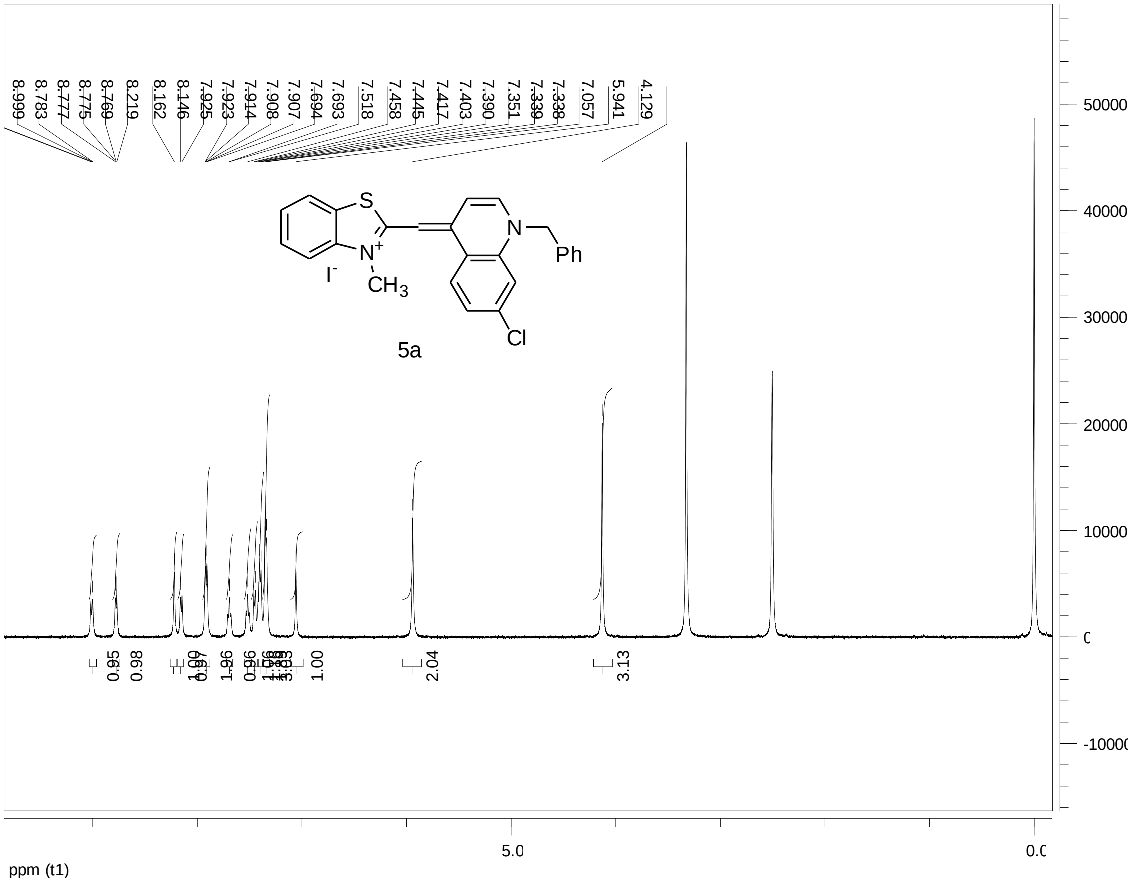

Figure S6: ^{1}H NMR spectrum of **5a**.

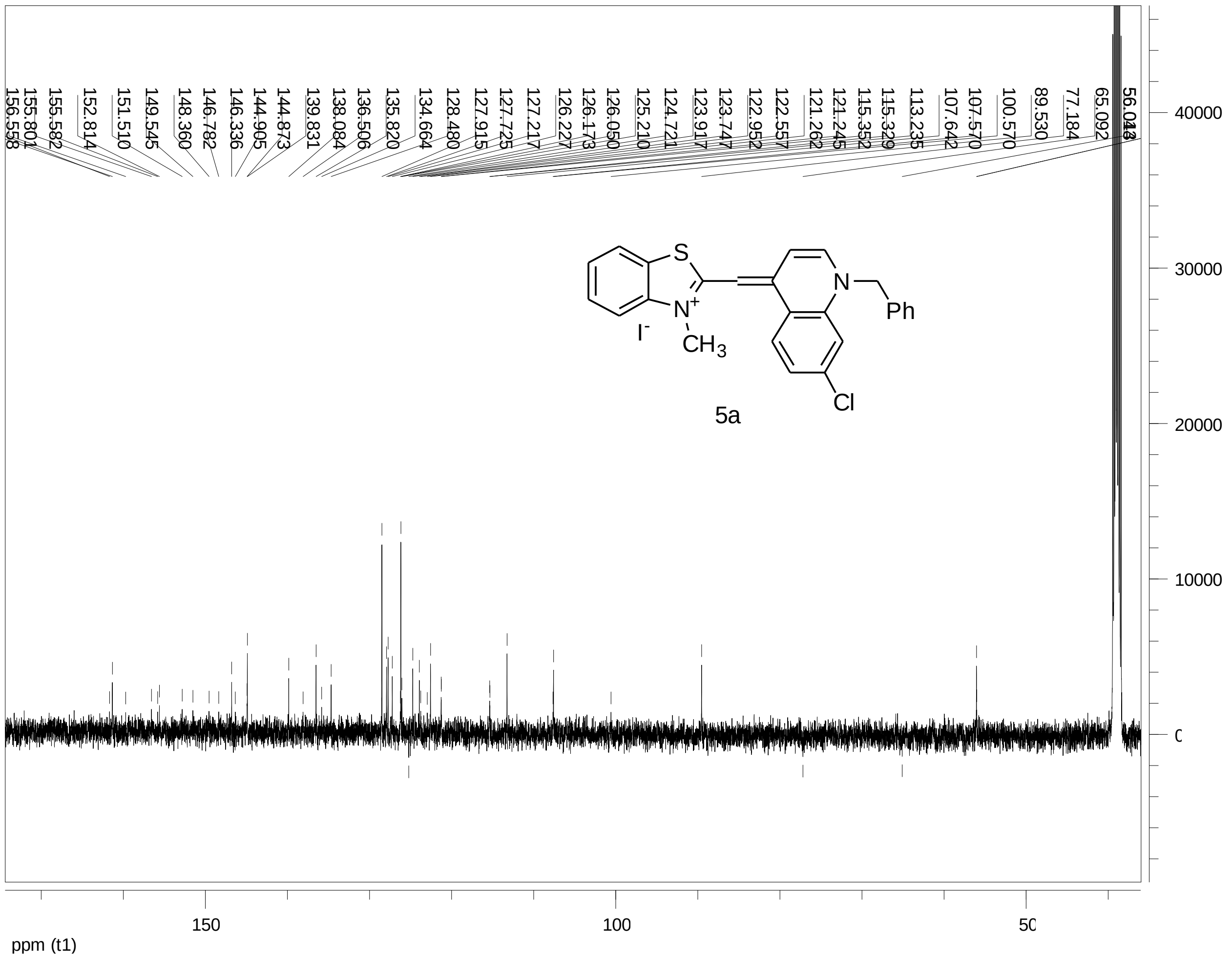

Figure S7: DEPT135 spectrum of **5a**.

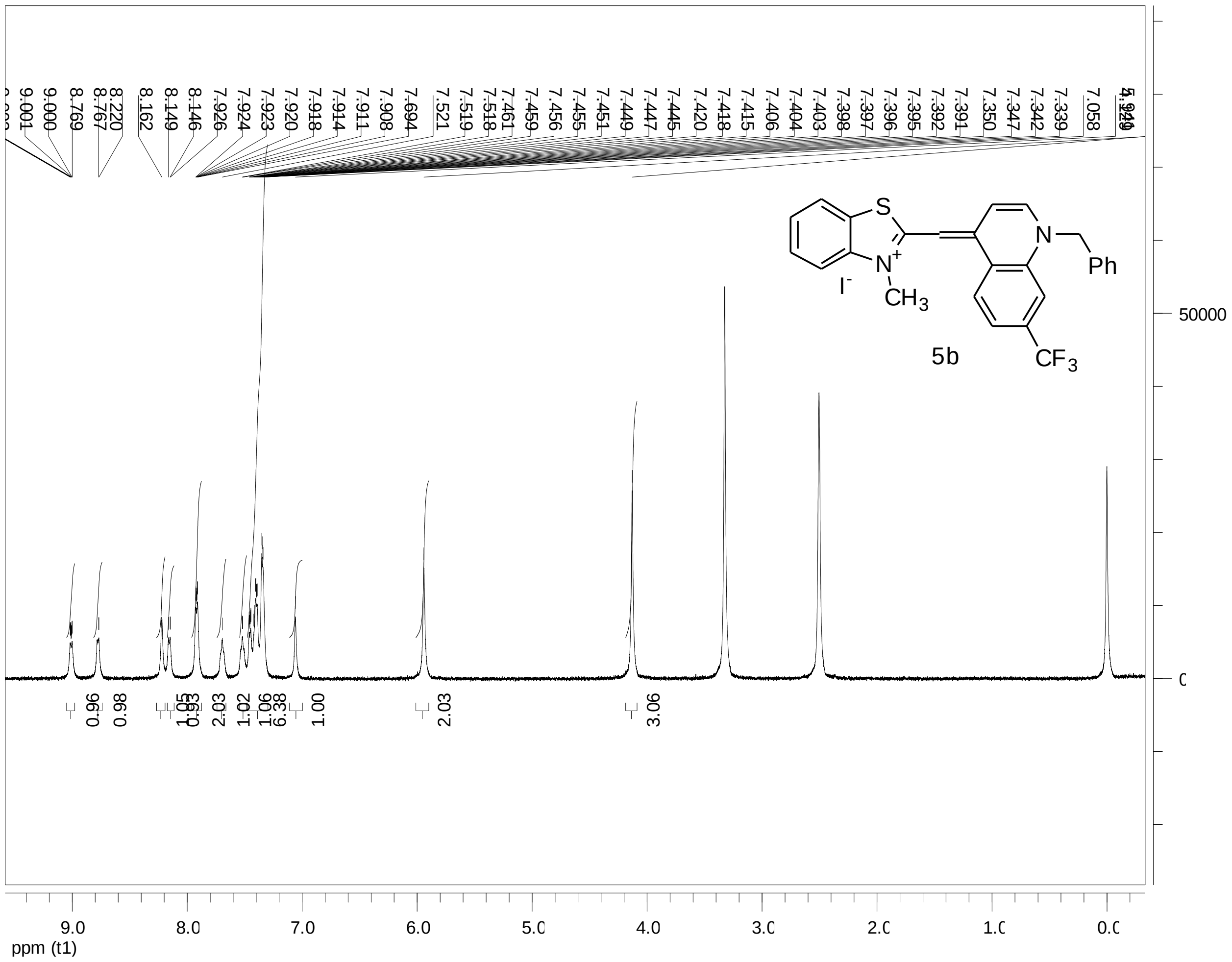

Figure S8: ^{1}H NMR spectrum of **5b**.

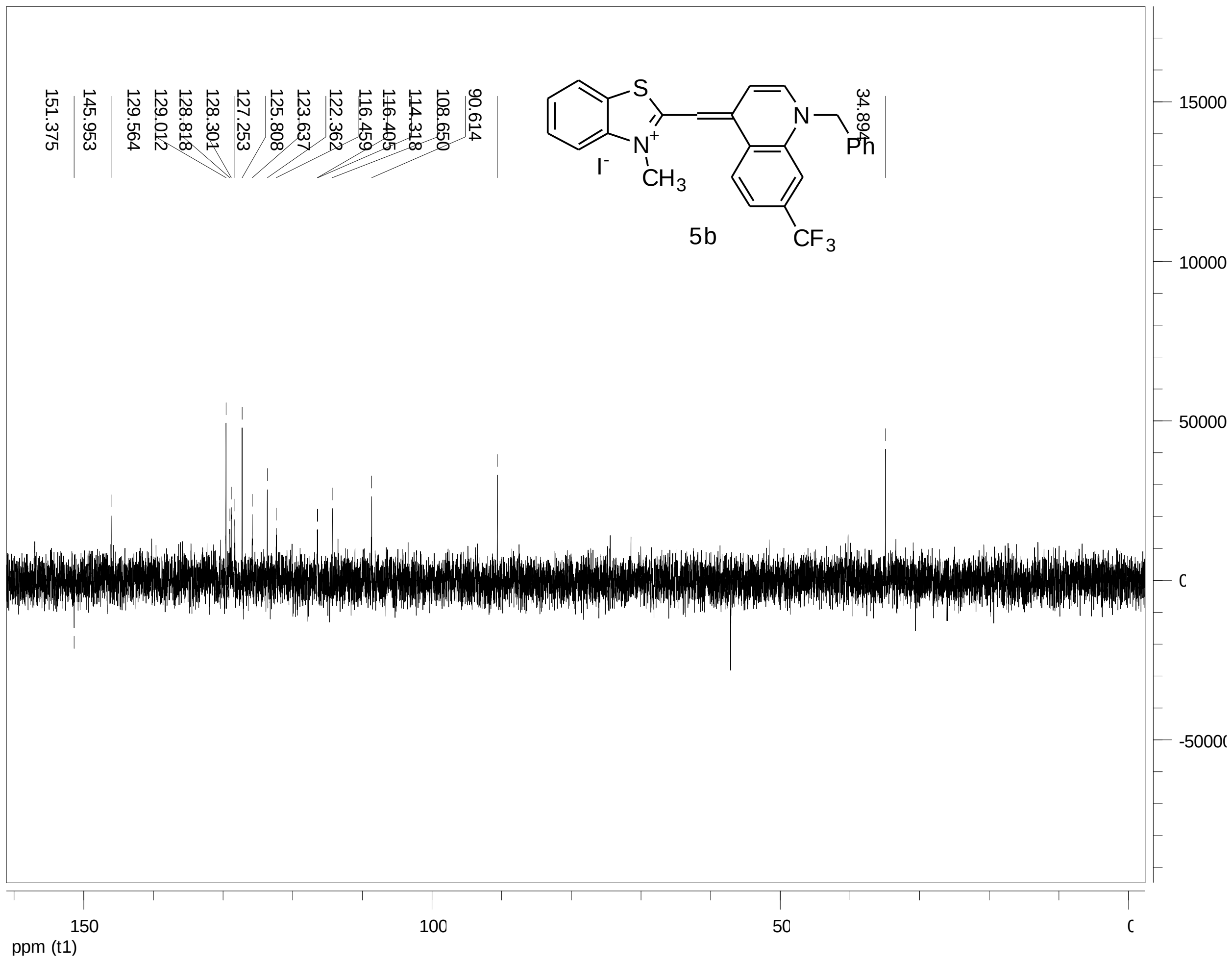

Figure S9: DEPT135 spectrum of **5b**.

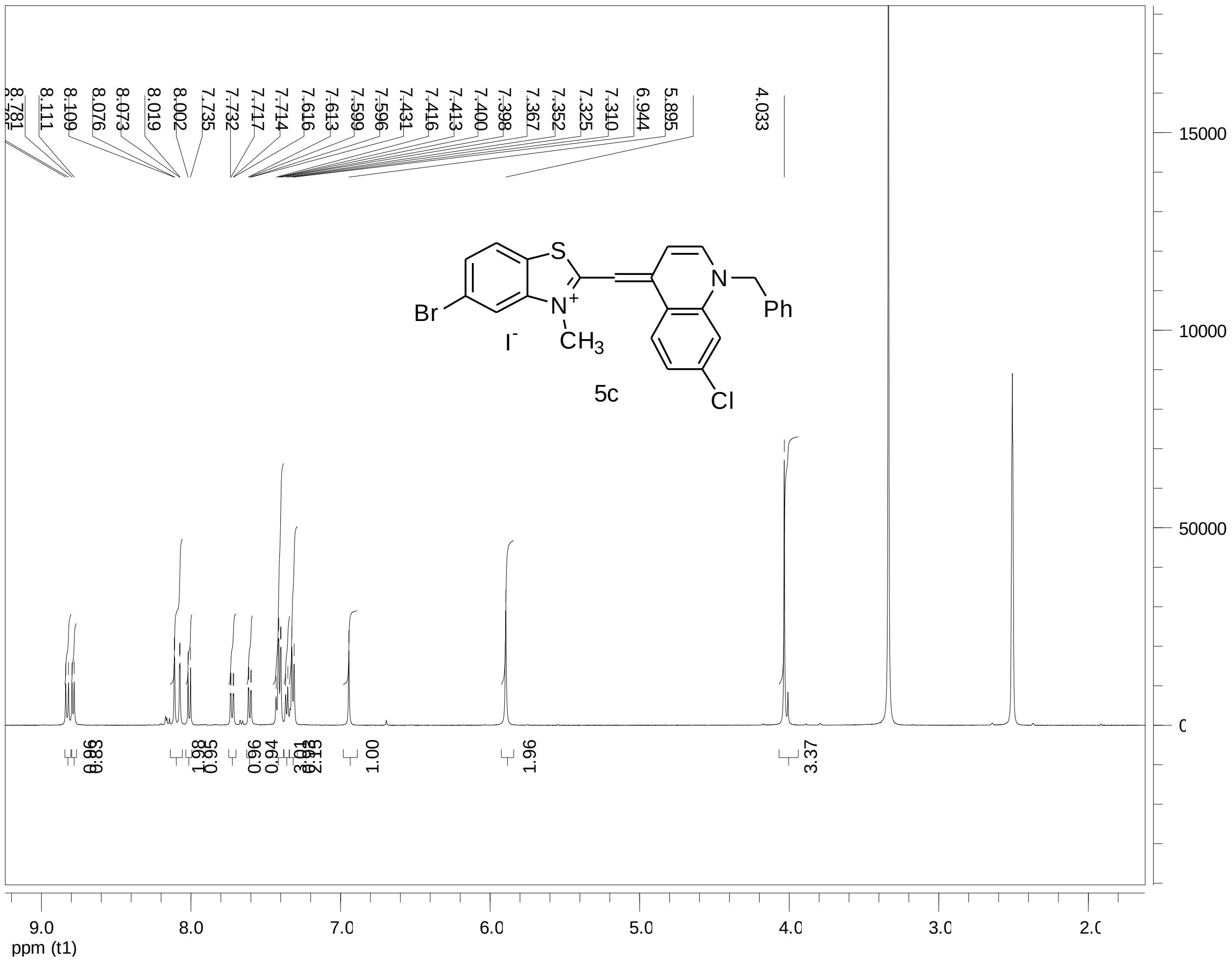

Figure S10: ^{1}H NMR spectrum of **5c**.

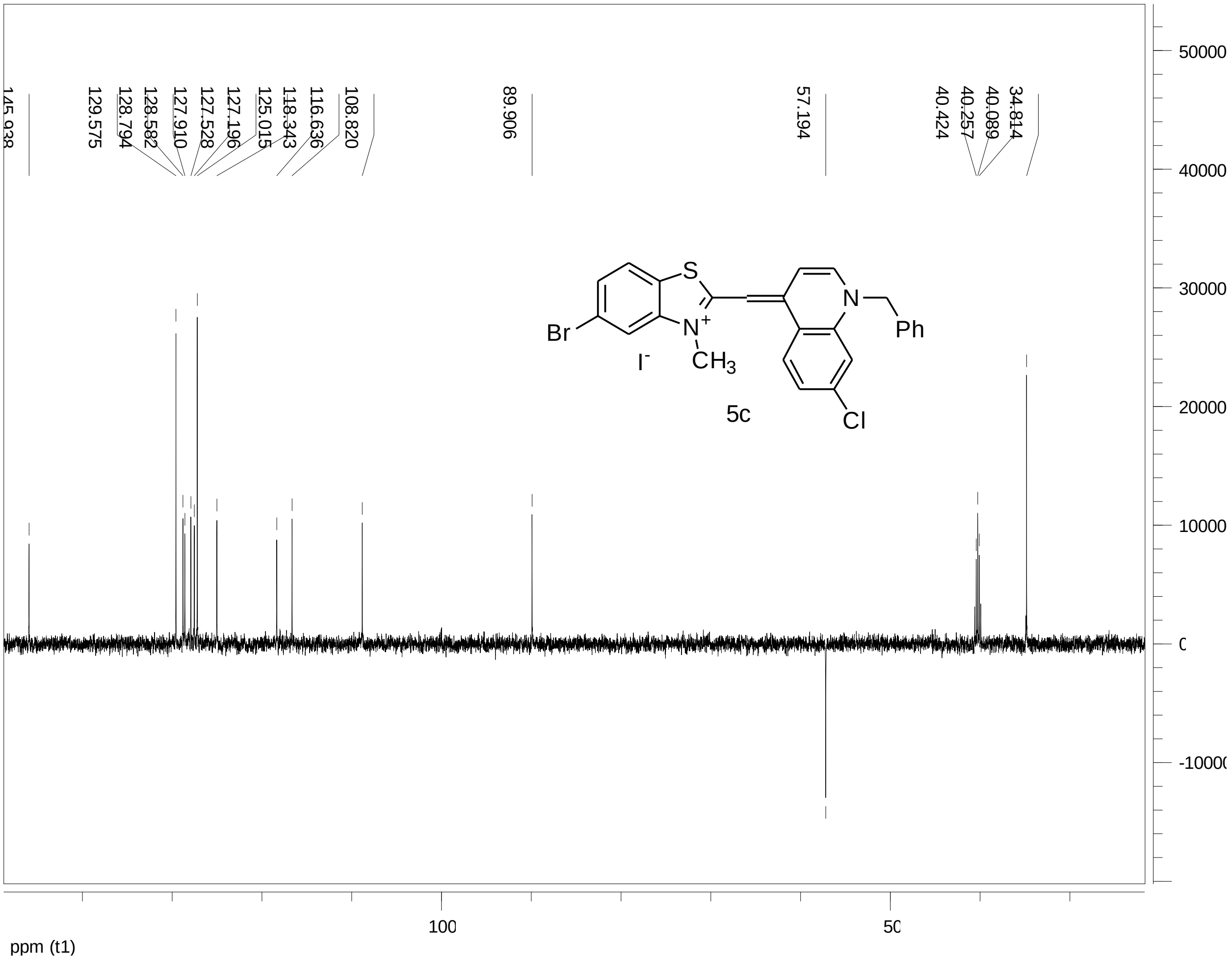

Figure S11: DEPT135 spectrum of **5c**.

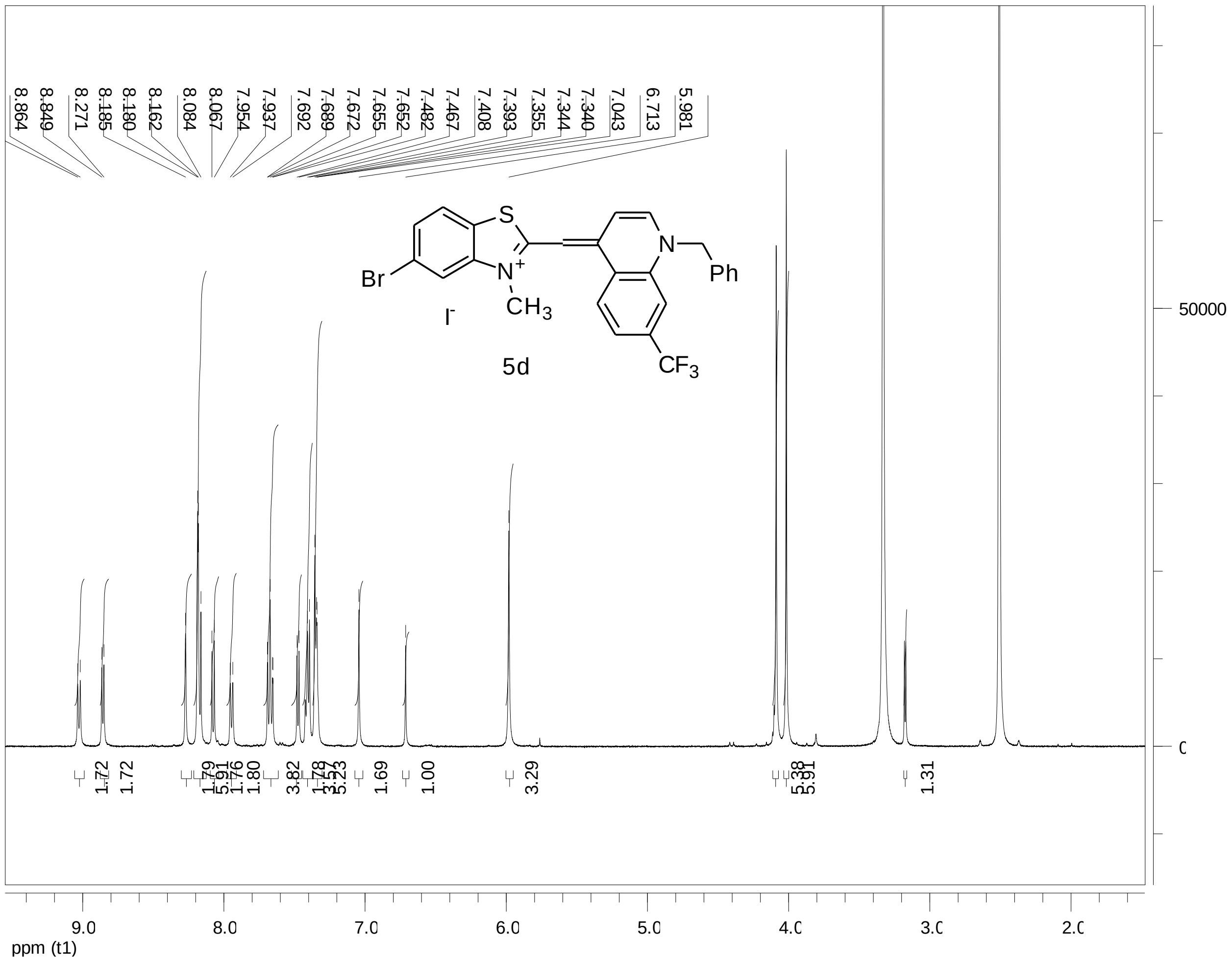

Figure S12: ^{1}H NMR spectrum of **5d**.

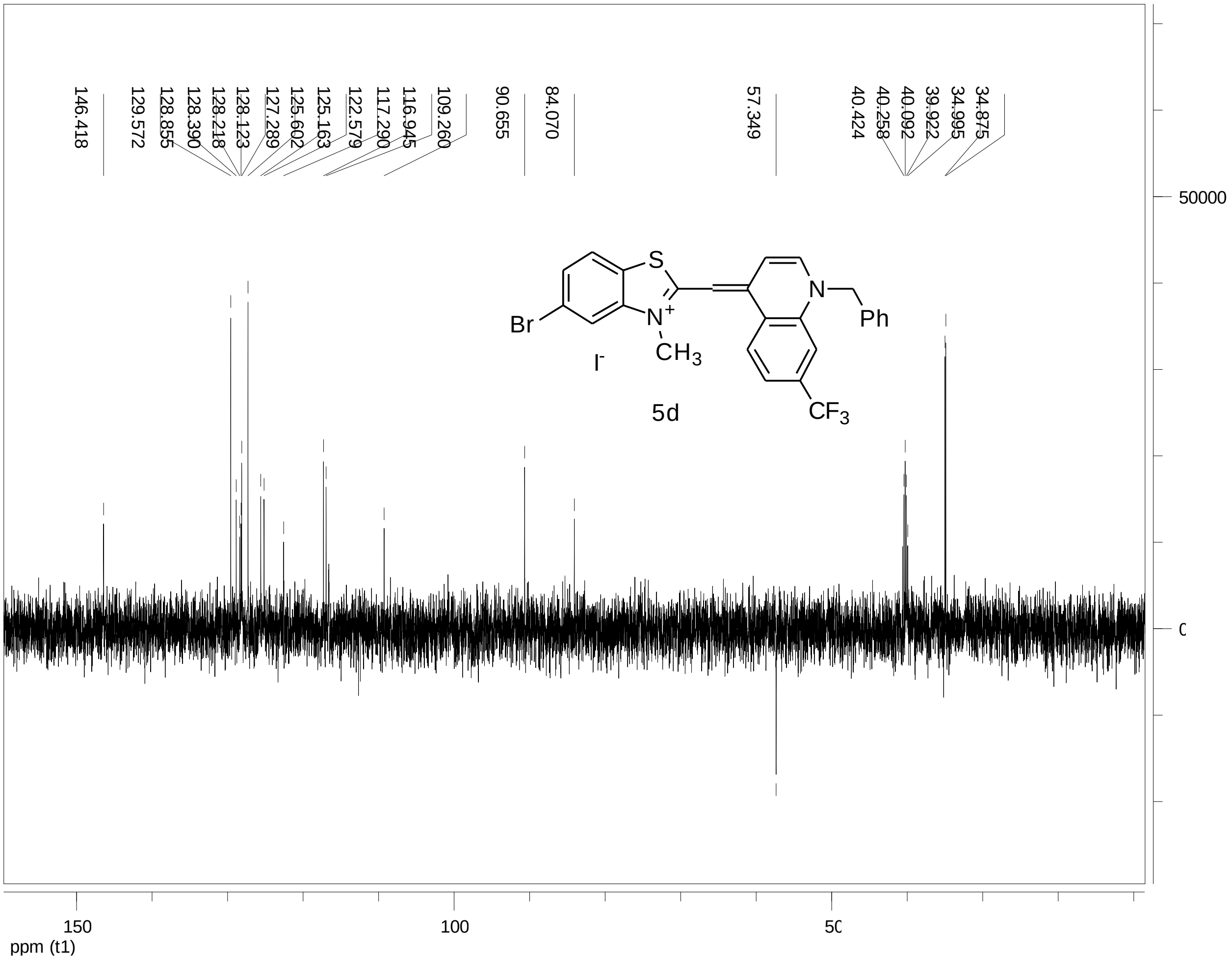

Figure S13: DEPT135 spectrum of **5d**.

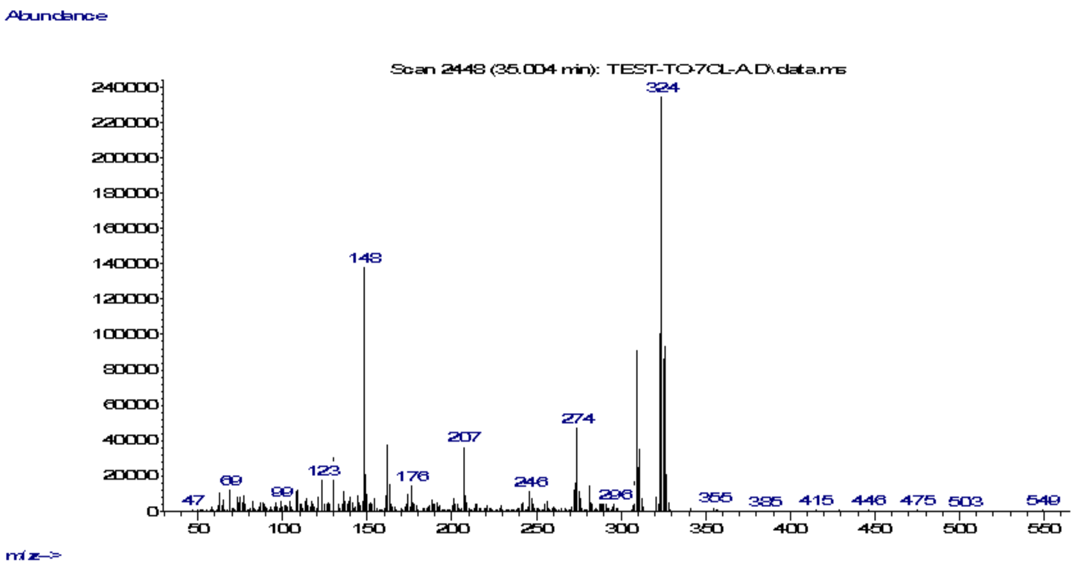

Figure S14: GC-MS spectrum of **TO-7Cl.**

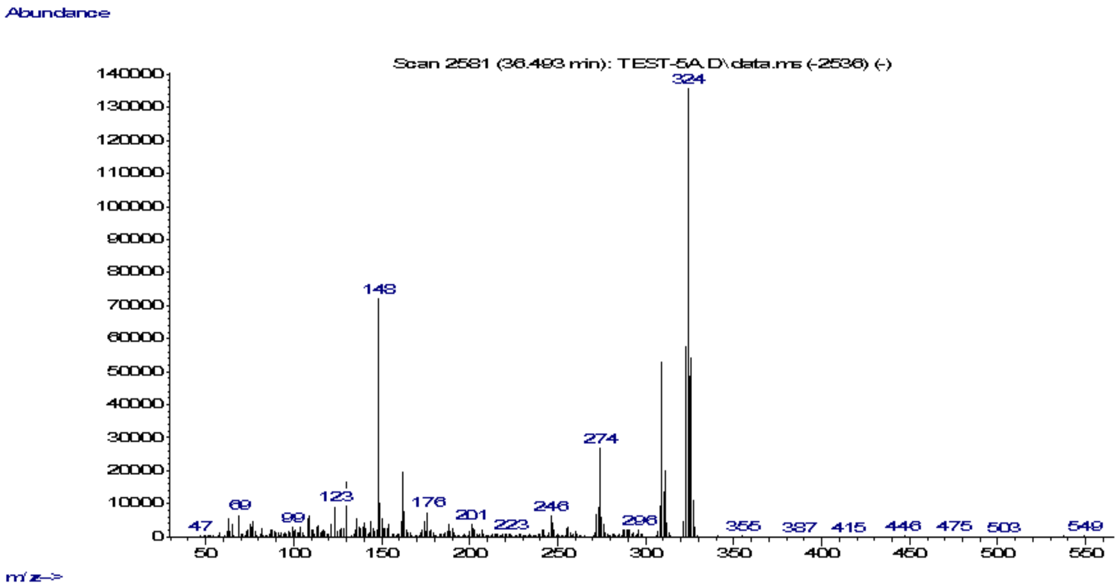

Figure S15: GC-MS spectrum of **5a.**

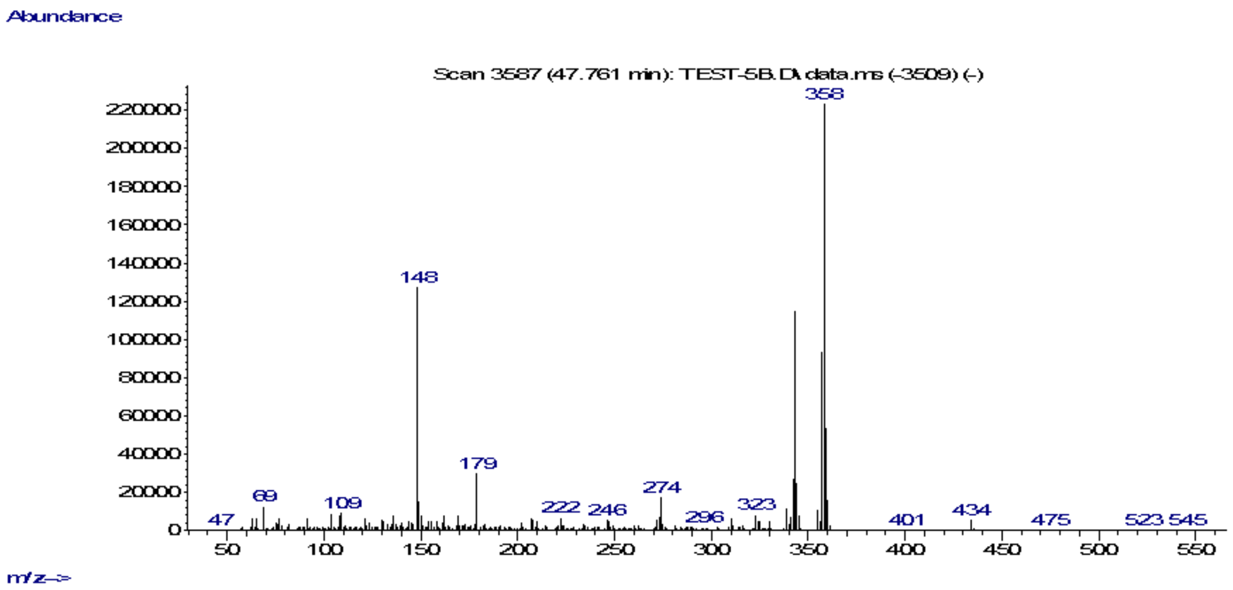

Figure S16: GC-MS spectrum of **5b.**

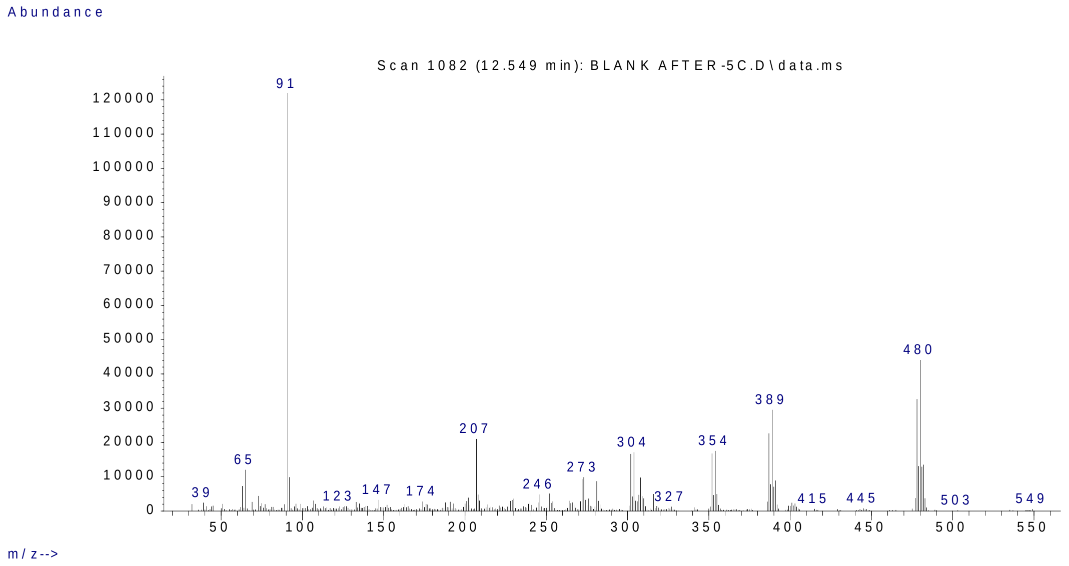

Figure S17: GC-MS spectrum of **5c.**

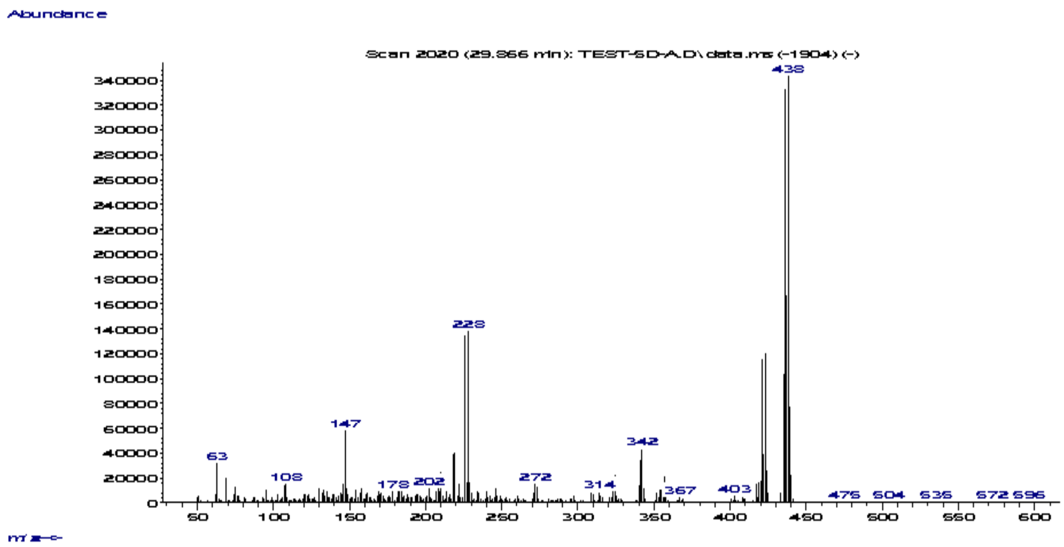

Figure S18: GC-MS spectrum of **5d.**

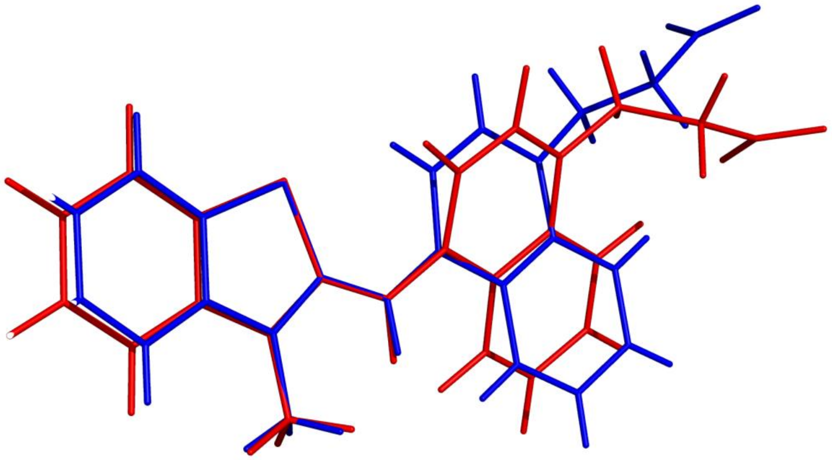

Figure S19: Overlay of 3-(4-((3-methyl-1,3-benzothiazol-2(3*H*)-ylidene)methyl)-quinolinium-1-yl)propanoate tetrahydrate (CSD ENTRY OVUJUL / CCDC number 739300) (blue) and B3LYP/6-31+G** optimized structure of 3-(4-((3-methyl-1,3-benzothiazol-2(3*H*)-ylidene)methyl)quinolinium-1-yl)propanoate (red).

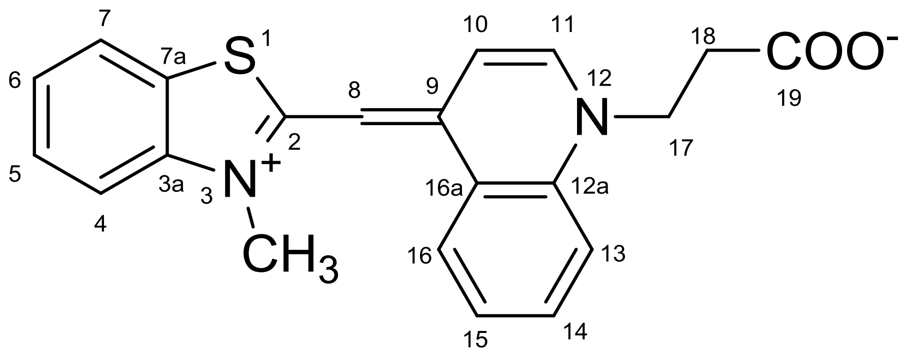

Figure S20: Labels assigned to selected atoms in the structure from ref. [S1].

Table S1: Experimental structural data (selected bond lengths and angles) from ref. [S1] compared to the B3LYP/6-31+G** calculated values for the fully optimized structure of 3-(4-((3-methyl-1,3-benzothiazol-2(3*H*)-ylidene)methyl)quinolinium-1-yl)propanoate without crystallization water molecules.

	**Calculated**	**Experimental (ref. [S1])**	**Exp. – Calc.**
Bond distances (Å)			
S1-C2	1.77	1.74	-0.03
C2-N3	1.38	1.37	-0.01
N3-C3a	1.40	1.39	-0.01
C3a-C4	1.40	1.40	0.00
C4-C5	1.40	1.39	-0.01
C5-C6	1.40	1.39	-0.01
C6-C7	1.40	1.38	-0.02
C7-C7a	1.39	1.39	0.00
C7a-S1	1.76	1.73	-0.03
C2-C8	1.38	1.39	0.01
C8-C9	1.43	1.40	-0.03
C9-C10	1.41	1.40	0.00
C10-C11	1.38	1.38	-0.01
C11-N12	1.34	1.34	0.00
N12-C12a	1.39	1.39	0.00
C12a-C13	1.41	1.40	-0.02
C13-C14	1.38	1.37	-0.01
C14-C15	1.41	1.41	0.00
C15-C16	1.38	1.35	-0.03
C16-C16a	1.42	1.42	0.00

C16a-C9	1.45	1.46	0.01
N12-C17	1.49	1.50	0.00
C17-C18	1.54	1.50	-0.04
C18-C19	1.58	1.54	-0.05
Dihedral angles (°)			
S1-C2-C8-C9	7.9	0.6	-7.30
C2-C8-C9-C10	19.2	0.7	-18.50

[S1]. Fei, X., Gu, Y., Lan, Y. et al., J. Chem. Crystallogr. (2011) 41: 1232. https://doi.org/10.1007/s10870-011-0080-0, Synthesis and Crystal Structure of Thiazole Orange Derivative

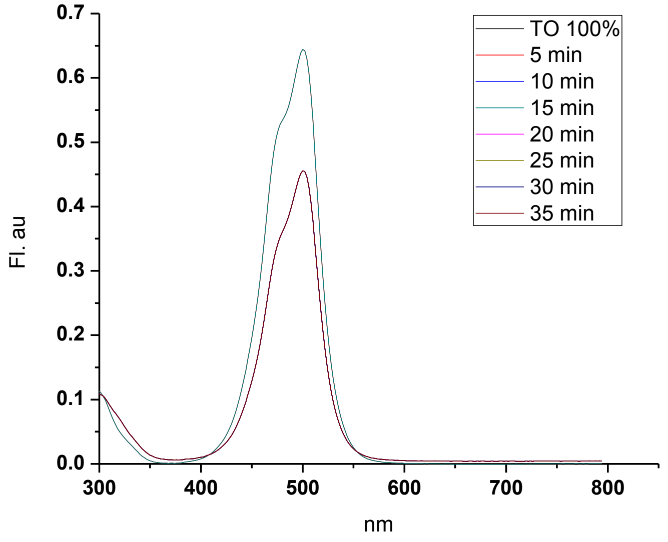

Figure S21: Photostability of **TO** measured by a continuous wave frequency doubled DPSS laser, operating at λ = 532 nm.

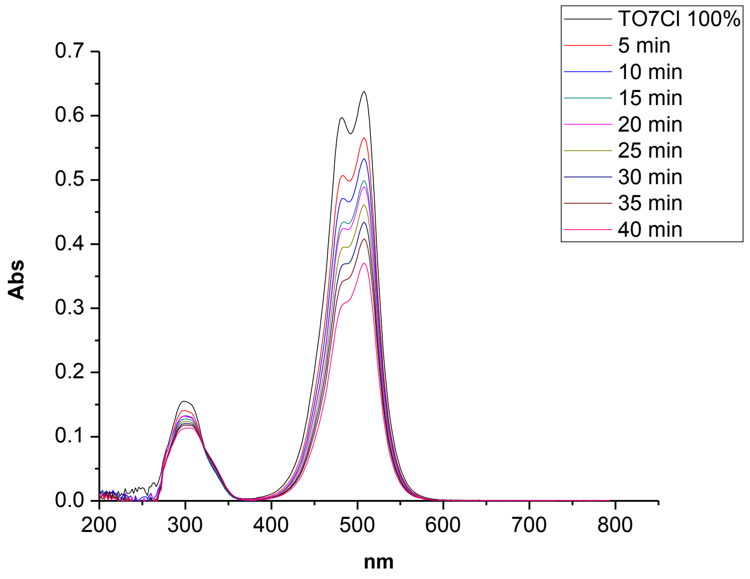

Figure S22: Photostability of **TO-7Cl** measured by a continuous wave frequency doubled DPSS laser, operating at λ = 532 nm.

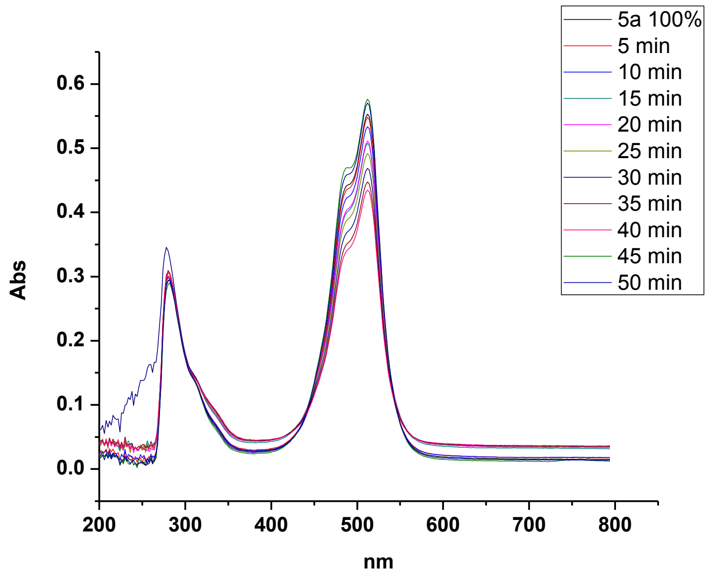

Figure S23: Photostability of **5a** measured by a continuous wave frequency doubled DPSS laser, operating at λ = 532 nm.

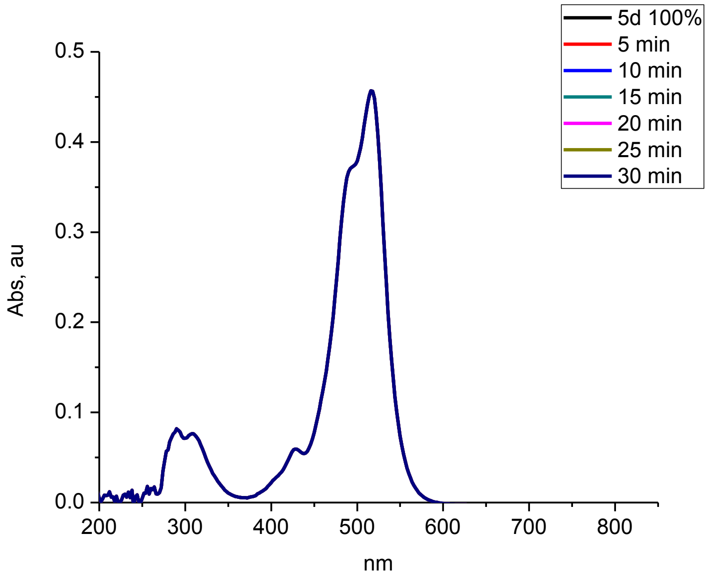

Figure S24: Photostability of **5d** measured by a continuous wave frequency doubled DPSS laser, operating at λ = 532 nm.
